# Vocal practice regulates singing activity–dependent genes underlying age-independent vocal learning in songbirds

**DOI:** 10.1371/journal.pbio.2006537

**Published:** 2018-09-12

**Authors:** Shin Hayase, Hongdi Wang, Eri Ohgushi, Masahiko Kobayashi, Chihiro Mori, Haruhito Horita, Katsuhiko Mineta, Wan-chun Liu, Kazuhiro Wada

**Affiliations:** 1 Graduate School of Life Science, Hokkaido University, Sapporo, Hokkaido, Japan; 2 King Abdullah University of Science and Technology, Computational Bioscience Research Center, Thuwal, Saudi Arabia; 3 Department of Psychology, Colgate University, Hamilton, New York, United States of America; 4 Department of Biological Sciences, Hokkaido University, Sapporo, Hokkaido, Japan; 5 Faculty of Science, Hokkaido University, Sapporo, Hokkaido, Japan; Princeton University, United States of America

## Abstract

The development of highly complex vocal skill, like human language and bird songs, is underlain by learning. Vocal learning, even when occurring in adulthood, is thought to largely depend on a sensitive/critical period during postnatal development, and learned vocal patterns emerge gradually as the long-term consequence of vocal practice during this critical period. In this scenario, it is presumed that the effect of vocal practice is thus mainly limited by the intrinsic timing of age-dependent maturation factors that close the critical period and reduce neural plasticity. However, an alternative, as-yet untested hypothesis is that vocal practice itself, independently of age, regulates vocal learning plasticity. Here, we explicitly discriminate between the influences of age and vocal practice using a songbird model system. We prevented zebra finches from singing during the critical period of sensorimotor learning by reversible postural manipulation. This enabled to us to separate lifelong vocal experience from the effects of age. The singing-prevented birds produced juvenile-like immature song and retained sufficient ability to acquire a tutored song even at adulthood when allowed to sing freely. Genome-wide gene expression network analysis revealed that this adult vocal plasticity was accompanied by an intense induction of singing activity-dependent genes, similar to that observed in juvenile birds, rather than of age-dependent genes. The transcriptional changes of activity-dependent genes occurred in the vocal motor robust nucleus of the arcopallium (RA) projection neurons that play a critical role in the production of song phonology. These gene expression changes were accompanied by neuroanatomical changes: dendritic spine pruning in RA projection neurons. These results show that self-motivated practice itself changes the expression dynamics of activity-dependent genes associated with vocal learning plasticity and that this process is not tightly linked to age-dependent maturational factors.

## Author summary

How is plasticity associated with vocal learning regulated during a critical period? Although there are abundant studies on the critical period in sensory systems, which are passively regulated by the external environment, few studies have manipulated the sensorimotor experience through the entire critical period. Thus, it is a commonly held belief that age or intrinsic maturation is a crucial factor for the closure of the critical period of vocal learning. Contrary to this idea, our study using songbirds provides a new insight that self-motivated vocal practice, not age, regulates vocal learning plasticity during the critical period. To examine the effects of vocal practice on vocal learning, we prevented juvenile zebra finches from singing during the critical period by postural manipulation, which separated the contribution of lifelong vocal experience from that of age. When these birds were allowed to freely sing as adults, they generated highly plastic songs and maintained the ability to mimic tutored songs, as normal juveniles did. Genome-wide transcriptome analysis revealed that both juveniles and singing-prevented adults, but not normally reared adults, expressed a similar set of singing-dependent genes in a song nucleus in the brain that regulates syllable acoustics. However, age-dependent genes were still similarly expressed in both singing-prevented and normally reared adult birds. These results exhibit that vocal learning plasticity is actively controlled by self-motivated vocal practice.

## Introduction

Both human speech and birdsong are acquired through vocal learning [1,2]. This learning process is achieved through sensory learning to memorize model sounds and sensorimotor learning based on matching auditory inputs and motor output to the model sounds by iterative self-motivated practice of vocalization. However, neither humans nor songbirds maintain their vocal learning ability equally well during all phases of life; the ability is circumscribed by critical/sensitive periods for vocal learning. Although there exist abundant studies on the critical period of sensory system development regulated by environmental stimuli [3–5], the neural mechanisms underlying the critical period for sensorimotor learning, especially for vocal learning, are not well understood.

**Fig 1:**
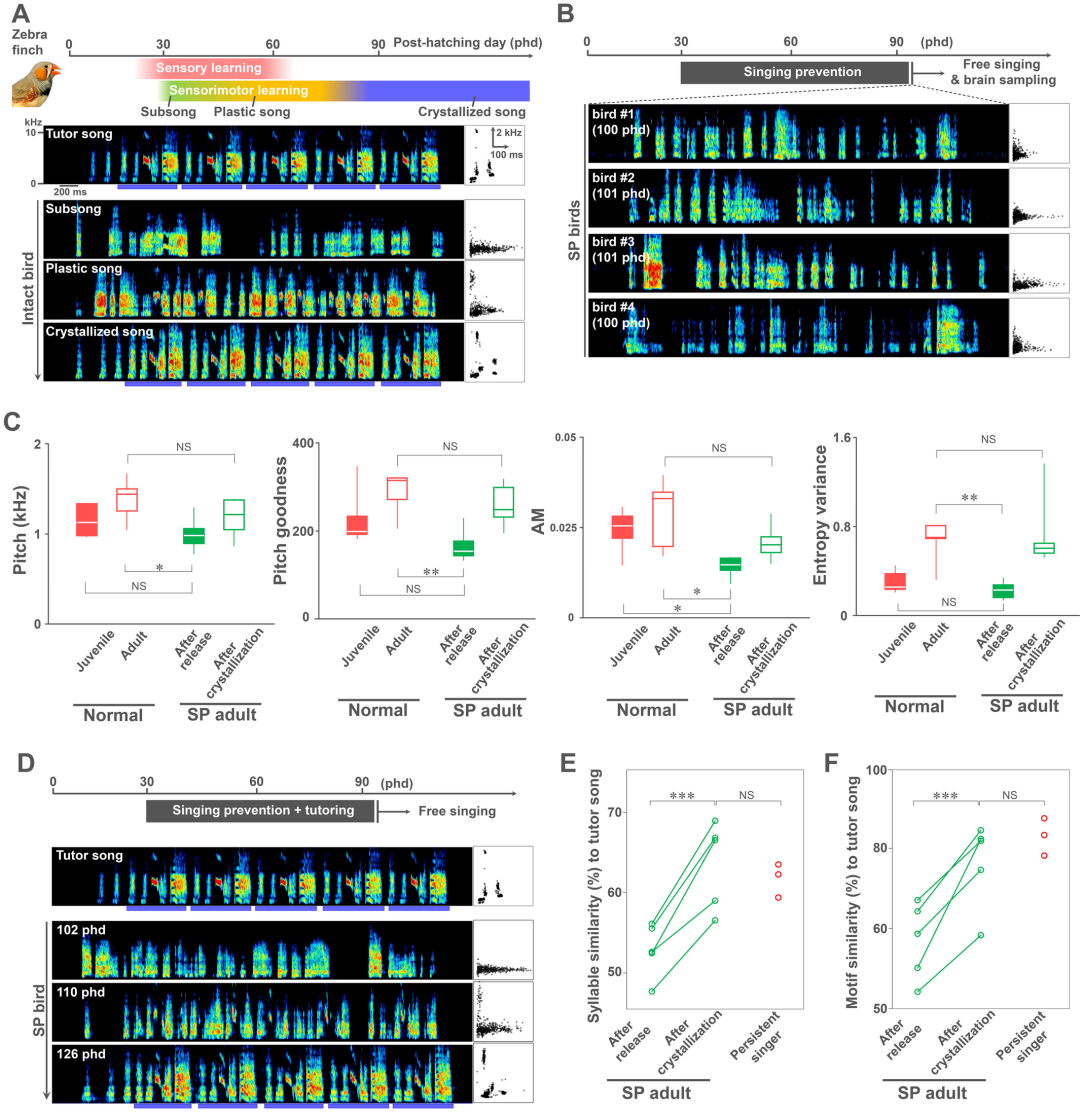
Singing experience–dependent regulation for the sensorimotor learning period of song acquisition in zebra finch. (A) Critical period of vocal learning (upper panel) and song development (lower panels) in the zebra finch. Blue bars in the lower panels represent the motif structure of crystallized song. Scatter plots indicate the distribution of 500 syllables (duration versus pitch). (B) Examples of songs of SP birds at 1–2 days after release at adult age (100–101 phd). (C) Comparison of syllable acoustic features, pitch, pitch goodness, AM, and entropy variance between normally reared juveniles (*n* = 5, 40–50 phd), normal adults (*n* = 5, 100–104 phd), SP birds after release (*n* = 5, 100–103 phd), and SP birds after song crystallization (*n* = 5, 119–126 phd). *p < 0.05, **p < 0.01; Welch’s *t* test. (D) Song development of an SP bird after release at adult age. Blue bars indicate the motif structure of crystallized song. (E, F) Comparison of mean syllable and motif similarities against tutor song between SP birds after release (*n* = 5, 100–103 phd) and after song crystallization (*n* = 5, 119–126 phd) and singing-persistent birds (*n* = 3, 101–105 phd. ***p < 0.005; Welch’s *t* test. ^NS^*p* > 0.05; unpaired *t* test. Supporting data can be found in S1 Data. AM, amplitude modulation; NS, not significant; phd, post hatching day; SP, singing-prevented.

The songbird is highly advantageous for studying the neural substrate of vocal learning and its critical period. The critical period of song learning includes 2 phases, the sensory and sensorimotor learning phases (Fig 1A). In the sensory learning phase, juveniles acquire sensory memories of song by listening to adult birds’ songs, which serve as a template to imitate. The sensorimotor learning phase starts with the generation of soft and highly variable syllables, called “subsong.” Thereafter, birds start producing “plastic song,” characterized by the gradual inclusion of recognizable yet variable syllables. At the end of the learning process, the song is crystallized with acoustically and sequentially stable syllable patterns (“crystallized song”). In a closed-ended vocal learner songbird, like the zebra finch, the time window of the sensorimotor learning phase lasts 2 months, beginning in juveniles at 30–45 post hatching day (phd) and ending in adulthood at 90–100 phd with the production of crystallized motif song patterns that are then maintained throughout life (Fig 1A). During the sensorimotor learning phase, zebra finches produce approximately 1,000 song renditions in a day through self-motivated vocal practice [6–8].

**Fig 2:**
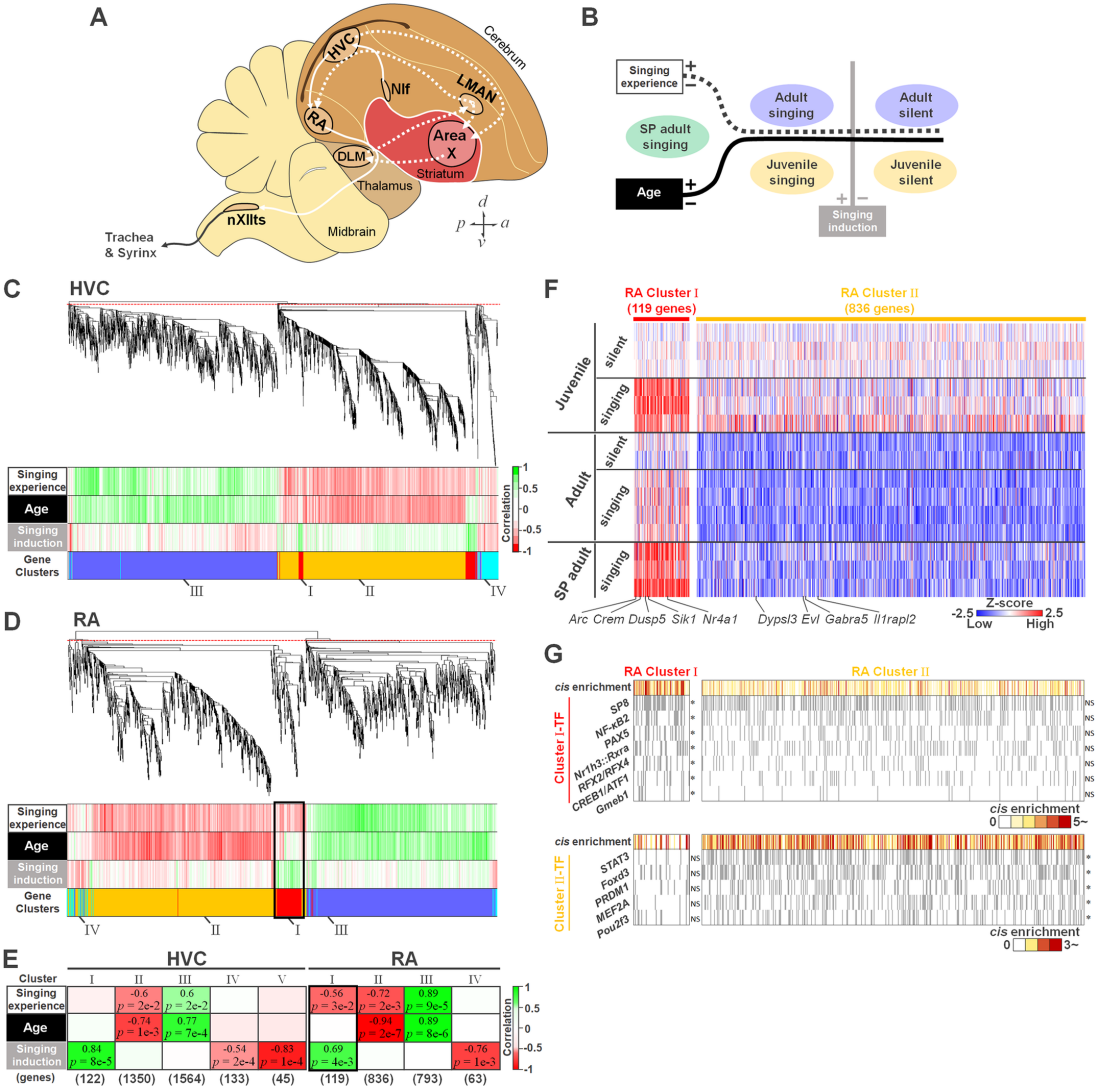
Cumulative singing experience regulates a cluster of singing activity–dependent genes in RA. (A) Schematic showing selected song-control regions and connections in the songbird brain. The posterior motor pathway and the anterior cortical-basal ganglia-thalamic circuit (anterior forebrain pathway) are represented as solid and dotted white lines, respectively. HVC used as a proper name; Area X = Area X of the striatum; nXIIts = tracheosyringeal part of the hypoglossal nucleus. (B) Sampling conditions for RNA-seq to extract transcriptome information on singing experience, age, and singing induction. (C, D) Upper panels: dendrogram of average linkage hierarchical clustering of differentially regulated genes in HVC (C) and RA (D) (3,214 and 1,811 genes, respectively). The red dotted line indicates the height at which the tree was cut. Lower panels: correlation heat maps between gene expression levels and each parameter: singing experience, age, or singing induction. Colored bands indicate positive (green) and negative (red) correlations. (E) Regulation relationships of gene clusters in HVC and RA for singing experience, age, and singing induction. Heat colors show correlations with parameters for each gene cluster. *P* values in each cell; student’s asymptotic *P* value. (F) Heat maps of Z scores of RA Cluster I and II genes (119 and 836 genes, respectively), normalized by average expression value of each gene at juvenile silent condition. (G) *Cis*-enrichment analyses of RA Cluster I and II genes. Each gray bar represents a predicted TF binding site. Supporting data can be found in S2 Data for panels C–G. DLM, dorsal lateral nucleus of the medial thalamus; LMAN, lateral magnocellular nucleus; NIf, interfacial nucleus of the nidopallium; RA, robust nucleus of the arcopallium; RNA-seq, RNA sequencing; SP, singing-prevented; TF, transcription factor.

Neuronal activity itself causes genetic responses in the brain. These activity-dependent genes either directly or indirectly influence the physiological function and structural maturation of neural circuits as genetic regulators for long-term neuronal plasticity [9–11]. Singing behavior also induces a set of activity-dependent genes in specialized brain regions, called the song nuclei (Fig 2A) [12–15]. The song nuclei are interconnected to form neural pathways for vocal learning and production [1,16]. Some of the singing activity–dependent genes are differentially regulated in the song nuclei between juvenile and adult stages [13,17–19]. However, a large variety of genes are developmentally regulated as age-related genes during the critical period of vocal learning in the song nuclei [20–22]. These studies suggest that the differentially regulated genes during the sensorimotor learning phase could be crucial molecules for modulation of vocal learning plasticity.

In this study, we investigated first how self-motivated vocal practice influences song maturation and second whether such practice modulates vocal learning plasticity during the sensorimotor learning phase in the zebra finch. Using a reversible singing prevention paradigm, we found that cumulative singing practice itself is an essential regulator of vocal learning plasticity: singing-prevented (SP) birds retained the ability to imitate a memorized tutored song into adulthood, well beyond the critical period of the sensorimotor learning phase. This age-independent sensorimotor learning was accompanied by significant changes in the expression of singing activity–dependent genes—but not age-related genes—in the projection neurons of song nuclei RA. Furthermore, the number of dendritic spines of the robust nucleus of the arcopallium (RA) was also affected by cumulative singing practice. These results show that self-motivated practice itself changes the expression dynamics of activity-dependent genes associated with vocal learning plasticity and that this process is not completely determined by age-dependent maturational factors.

## Results

### Prevention of vocal practice extends the sensorimotor learning period in the zebra finch

The zebra finch (*Taeniopygia guttata*) is termed a closed-ended learner songbird because they can only learn during a critical period and subsequently produce a stereotyped song (Fig 1A)[23,24]. To elucidate the importance of self-motivated vocal practice to song development during the sensorimotor learning phase in contrast to age itself, we prevented juvenile zebra finches from singing before initiation of their first song (approximately 30 phd) until adulthood (91–133 phd, mean = 101.6 phd) by postural manipulation (Fig 1B and S1 Fig). The postural manipulation was performed by attaching a custom-made weight on the neck of juvenile birds only during daylight hours, based on a modified method that uses weights to manipulate singing [25]. The weight shifted their posture slightly toward an inferior position (approximately at 0.5–1.5 cm lower than normal height); the weight was supported by the floor of the cage (i.e., not carried by the bird’s neck). Although this manipulation prevented singing, the birds were exposed to a tutor song produced by their biological fathers and could still freely generate daily behaviors, such as drinking, eating, and producing non-song-related vocalization, like calls. The weight was detachable and daily adjusted for each bird (up to 16.5–24.0 g) so that it was at the threshold of preventing singing without adversely affecting the bird’s health or bodily growth. To account for the overall health of the animals, body weights were regularly monitored. Body weight and acoustic features of call vocalizations were not significantly different between normally reared and SP birds (*P* > 0.05, 1-way ANOVA with Bonferroni correction for body weight; *P* > 0.05, Welch’s *t* test for call acoustics) (S1 Fig), demonstrating no adverse effects on the growth of the peripheral vocal organs, such as the syrinx.

Singing prevention was highly effective: normal birds produce over 60,000 song bouts during the sensorimotor learning period (approximately 1,000 bouts/day × 60 days) [6], while the singing prevention birds produced less than 0.1% of this output (24–485 song bouts, mean = 279.0 bouts). When the birds were released from singing prevention at adulthood, they produced “age-unmatched” immature songs with highly variable acoustics and sequence of syllables, i.e., subsong or early plastic-like song (Fig 1B). To quantify the immaturity of song quality in SP birds, we calculated the values of pitch, pitch goodness, and amplitude modulation (AM) and the entropy variance of syllables as acoustic parameters [26,27] and motif consistency as a parameter of song sequence [20]. In all parameters, songs of the SP birds were similar to the subsong/early plastic song produced by normal juveniles (Fig 1C). Despite the manipulation, in 3 of 24 birds, the weight-based postural manipulation did not affect singing practice, i.e., the birds persistently continued singing (more than 10,000 bouts of total singing). Even under the postural manipulation condition, the persistent singers developed crystallized songs with the typical motif structure and copied song traits from their tutors (S1 Fig), indicating that iterative singing experience per se but not the experimental handling influences song development and learning.

In adult SP birds producing immature-like plastic songs, we investigated whether or not they also retained vocal learning plasticity to mimic the tutor songs that they had already memorized. We found that SP birds quickly crystallized structured songs within 4 weeks after release at adulthood (Fig 1D and S2 Fig), which was less than half the duration of the normal sensorimotor learning period in the zebra finch. Moreover, the syllables produced by the SP birds, despite being crystallized so rapidly, had the same acoustic traits that were observed in unmanipulated adults (Fig 1C). In addition, the SP adult birds did not only develop species-typical crystalized songs, but they also mimicked their tutor songs at the levels of both syllable acoustics and sequence (motif) features. Comparison between the songs produced at 1–2 days after release (phd 101–103) and ones after 3–4 weeks (phd 120–126) revealed a significant increase of syllable and motif similarity scores toward their tutor songs (paired *t* test: syllable, *t*(4) = 7.7, *P* = 0.0015; motif, *t*(4) = 5.8, *P* = 0.0044) (Fig 1E and 1F). A subset of the SP adults near-perfectly mimicked the phonology and sequence order of all syllables of the memorized tutor song (S2 Fig). Consistently, the SP birds showed a similar imitation accuracy of their acquired songs against tutor songs as did the birds that persistently continued singing (Welch’s *t* test: syllable, *t*(6) = 0.68, *P* = 0.52; motif, *t*(6) = 1.2, *P* = 0.27) (Fig 1E and 1F). These results indicate that cumulative singing experience (i.e., vocal practice) acts as an age-independent sensorimotor learning mechanism in the zebra finch.

### Cumulative singing experience, but not age, regulates a novel set of singing activity–dependent genes

We conducted a genome-wide gene expression network analysis comparing juvenile, adult, and SP adult (1–2 days after release from the postural manipulation) birds to elucidate the transcriptional impacts of cumulative singing experience versus age in the brain regions. The song nuclei are interconnected to form 2 functional circuits: one being the vocal motor pathway (VMP) and another forming the anterior forebrain pathway (AFP), a cortical–basal ganglia–thalamic loop (Fig 2A) [28–30]. For this purpose, we sampled laser microdissected tissues of 2 song nuclei HVC and RA in the VMP, which regulate syllable sequence and acoustics, respectively (Fig 2A and 2B). In 12,156 genes expressed in the telencephalon of the zebra finch, 3,214 and 1,811 genes were identified in HVC and RA, respectively, as genes that were differentially regulated by age and/or singing. For these genes, a weighted gene coexpression network analysis (WGCNA) identified 5 and 4 “Gene Clusters” in HVC and RA, respectively, as the coexpressed genes correlated with singing experience, age, and/or singing induction (Fig 2C–2E). Only one of the gene clusters, RA Gene Cluster I, met criteria that were significantly regulated by cumulative singing experience instead of age. This gene cluster contained 119 genes, including a novel set of singing activity–dependent genes (transcription factors/regulators: *Atf3*, *Crem*, *Nr4a1*, and *Irf8*; subunits of histone deacetylase complex: *Fam60a*; serine/threonine kinases: *Sik1* and *Sgk1*; and mitogen-activated protein (MAP) kinase phosphatases: *Dusp5* and *6*). In general, the expression of neuronal activity–dependent genes in song nuclei is regulated by singing rather than hearing [12,20]. RA Gene Cluster I was induced by diurnal acute singing. However, the singing-driven induction response was gradually attenuated as singing experience accumulated over time (Fig 2E and 2F), suggesting that the long-term cumulative experience of vocal practice progressively dampens the expression of singing activity–dependent genes as the sensorimotor learning phase progresses.

WGCNA further indicated that other gene clusters (Cluster II and III) were regulated by both singing experience and age (Fig 2C–2E). However, when comparing each individual gene expression of the clusters in the expression heat map, most of the genes in Cluster II and III were similarly expressed in both normally reared and SP birds as age-regulated genes (Fig 2F and S3 Fig), indicating that SP birds normally retain the developmental expression dynamics of age-regulated genes. In line with this, a *cis*-enrichment analysis revealed that differentially unique sets of transcription factors presumably bound the promoter regions of the coexpressed genes between different clusters. For an example, transcription factor families, including *Nf-κB*, *Creb/Atf*, *Sp*, *Rfx*, and *Pax*, were significantly enriched in putative proximal promoter regions (less than 1 kb from the transcription start site [TSS]) of the RA Cluster I genes but were not enriched in Cluster II genes (both Fisher’s exact test and G test, *p* < 0.05) (Fig 2G). These results suggest cumulative singing experience regulates the transcriptional of activity-dependent genes in RA.

**Fig 3:**
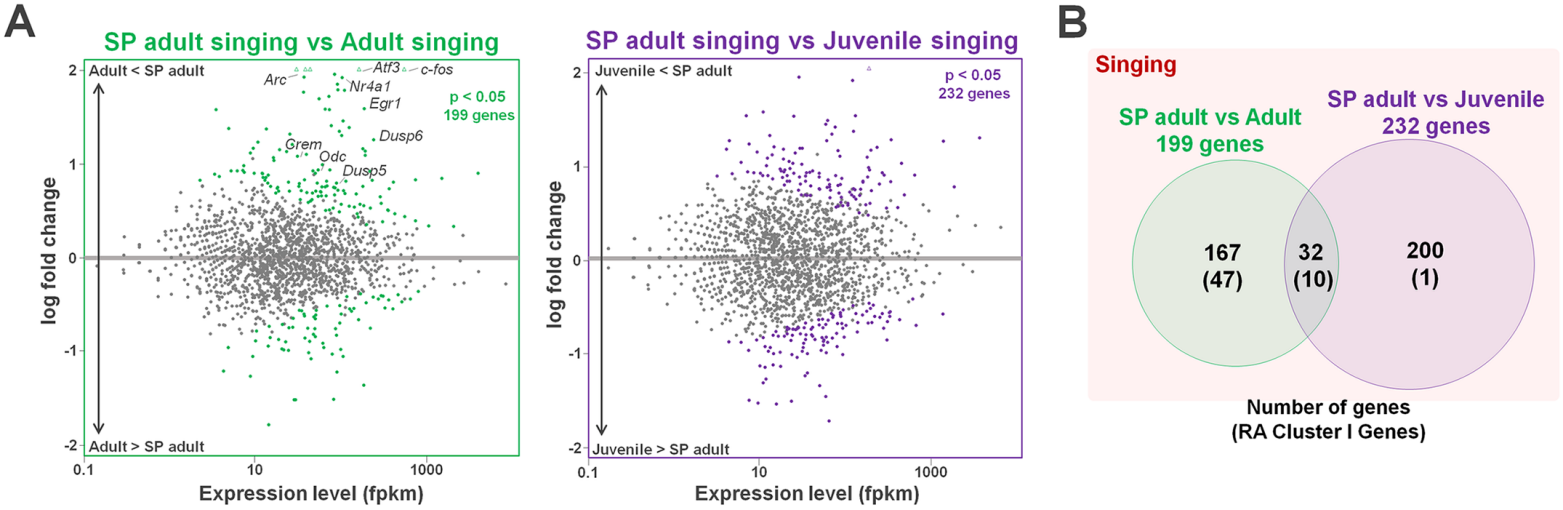
Direct comparison of the RA transcriptomes under singing condition between SP adult, normal adult, and juvenile groups. (A) MA plots indicate the expression of differentially regulated RA genes between SP adult singing versus normal adult singing (left) and between SP adult singing versus juvenile singing (right). (B) Number of genes compatibly identified as differentially regulated genes by DEseq2 and RA Cluster I genes by WGCNA. Supporting data can be found in S3 Data. fpkm, fragments per kilobase of exon per million mapped fragments; RA, robust nucleus of the arcopallium; SP, singing-prevented; WGCNA, weighted gene coexpression network analysis.

In addition, to ensure that the expression of RA Cluster I genes reflects cumulative singing experience but not age, we further performed direct comparisons of the RA transcriptomes between the SP adult singing versus the normal adult singing groups and between the SP adult singing versus the juvenile singing groups (Fig 3). As the result, a total 57 of 119 RA Cluster I genes were sorted into the differentially regulated genes between the SP adult versus the normal adult groups (Fig 3B; green) but few into the ones between the SP adult versus juvenile groups (Fig 3B; purple). This result reconfirms that the expression of singing activity–dependent genes in the RA Cluster I is modified by cumulative singing experience instead of age.

### Expression of cumulative singing experience–regulated genes in the song nuclei

**Fig 4:**
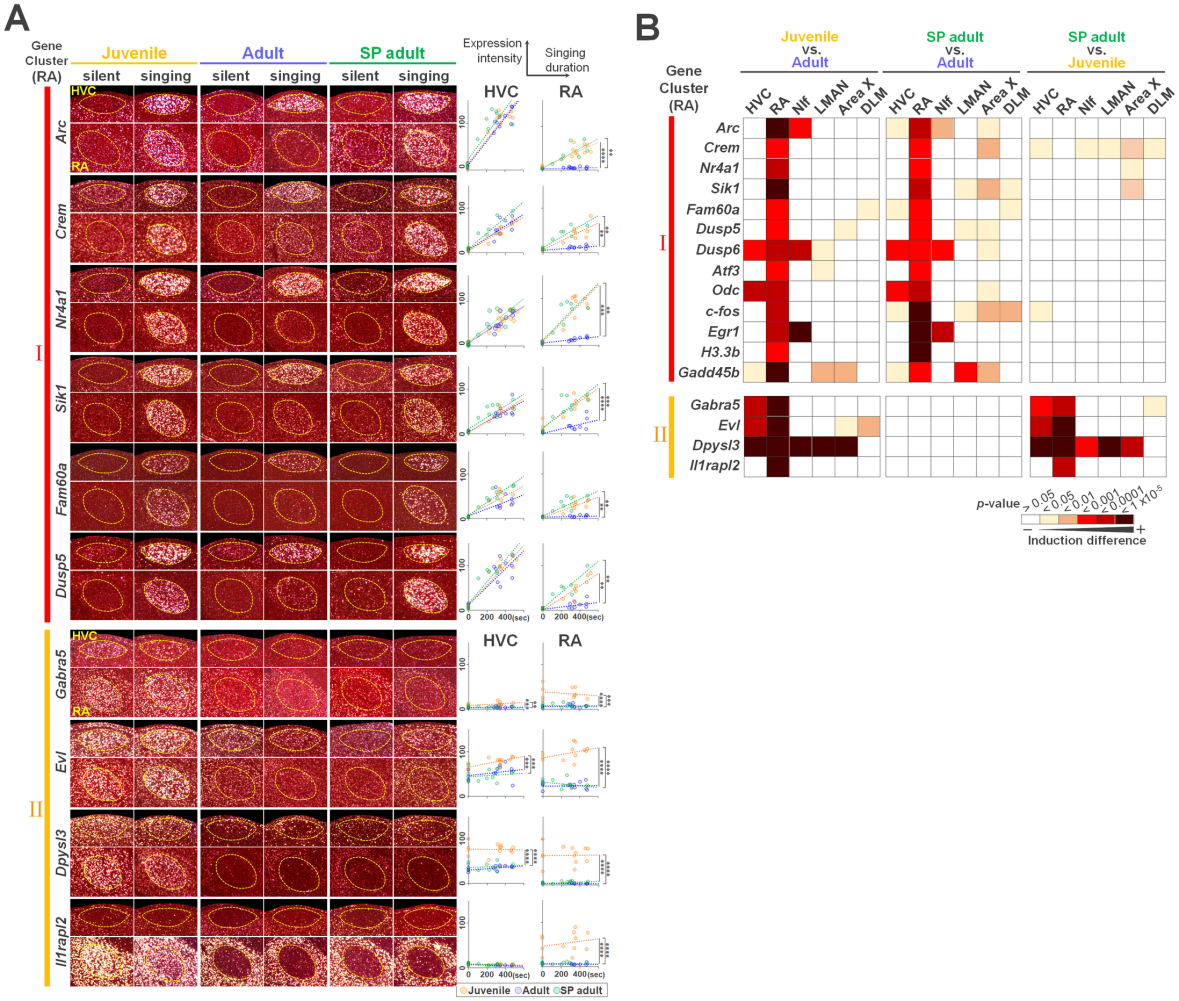
Cumulative singing experience–regulated gene expression in RA projection neurons between silent and singing condition. (A) RA Cluster I (*Arc*, *Crem*, *Nr4a1*, *Sik1*, *Dusp5*, and *Fam60a*) and II (*Gabra5*, *Evl*, *Dpysl3*, and *Il1rapl2*) gene expression in HVC and RA in juvenile, adult, and SP adult (1–2 days after release) birds. Right panels: induction intensity of the singing activity–dependent genes in juvenile (orange), adult (blue), and SP adult (green) birds in HVC and RA. The last 30 minutes of the singing duration of each bird is shown at the bottom. Lines represent linear approximation curve (*p < 0.01, **p < 0.001, ***p < 0.0001, ****p < 0.00001; ANCOVA with Bonferroni correction). (B) Heat maps showing induction differences of Cluster I and II genes in song nuclei between adult, juvenile, and SP adult (1–2 days after release) birds (ANCOVA with Bonferroni correction). Supporting data can be found in S4 Data. ANCOVA, analysis of covariance; DLM, dorsal lateral nucleus of the medial thalamus; LMAN, lateral magnocellular nucleus; NIf, interfacial nucleus of the nidopallium; RA, robust nucleus of the arcopallium; SP, singing-prevented.

To reveal the expression pattern of the cumulative singing experience–regulated genes in the entire song nuclei for vocal learning and production, we compared gene expression between silent and singing conditions in juveniles, adults, and SP adults (1–2 days after their release from the postural manipulation). We chose 13 genes: *Arc*, *Crem*, *Nr4a1*, *Sik1*, *Dusp5*, *Fam60a*, *Atf3*, *c-fos*, *Egr1*, *H3*.*3b*, *Gadd45b*, *Dusp6*, and *Odc*, which were independently identified as the cumulative singing experience–regulated genes by WGCNA (RA Cluster I genes in Fig 2) and the direct transcriptome comparison between normal and SP singing birds (Fig 3). All 13 tested genes were induced by singing and had a unique expression pattern in song nuclei between the 3 groups (Fig 4A, S4 and S5 Figs). In addition, singing activity–dependent gene expressions were differently regulated among song nuclei and also between the juvenile, adult, and SP adult groups. For example, *Arc* was consistently and intensely induced by singing in HVC, lateral magnocellular nucleus (LMAN), and Area X in all 3 groups. However, in RA, although juveniles and SP adult birds showed strong response of *Arc* expression after singing, the singing-driven induction was attenuated in normal adults (Fig 4A and S4 Fig). We therefore compared each gene induction after singing in each song nucleus between the 3 groups and then identified that RA is a major region that differently regulated the singing-driven induction response between the 3 groups (Fig 4B, S4 and S5 Figs). SP adults showed a striking resemblance to normal juveniles in the expression dynamics of the cumulative singing experience–regulated genes throughout song nuclei that was unlike normally reared adults (Fig 4B). Induction of 5 of 13 tested genes (*Arc*, *Fam60a*, *Dusp6*, *Odc*, and *Gadd45b*) was almost fully repressed in RA during adult singing, although singing evokes robust neuronal activity in RA in both juvenile and adult stages [31]. In contrast, a set of age-regulated genes identified as RA Gene Cluster II (including *Gabra5*, *Evl*, *Dpysl3*, and *Il1rapl2*) showed similar expression levels and patterns between SP and normal adults (Fig 4A and 4B and S6 Fig). These results indicate that SP birds selectively maintain juvenile-like expression for singing activity–dependent genes but express age-regulated genes in the entire song circuits similarly to untreated adults.

### Cumulative singing experience–mediated morphological changes in RA projection neurons

**Fig 5:**
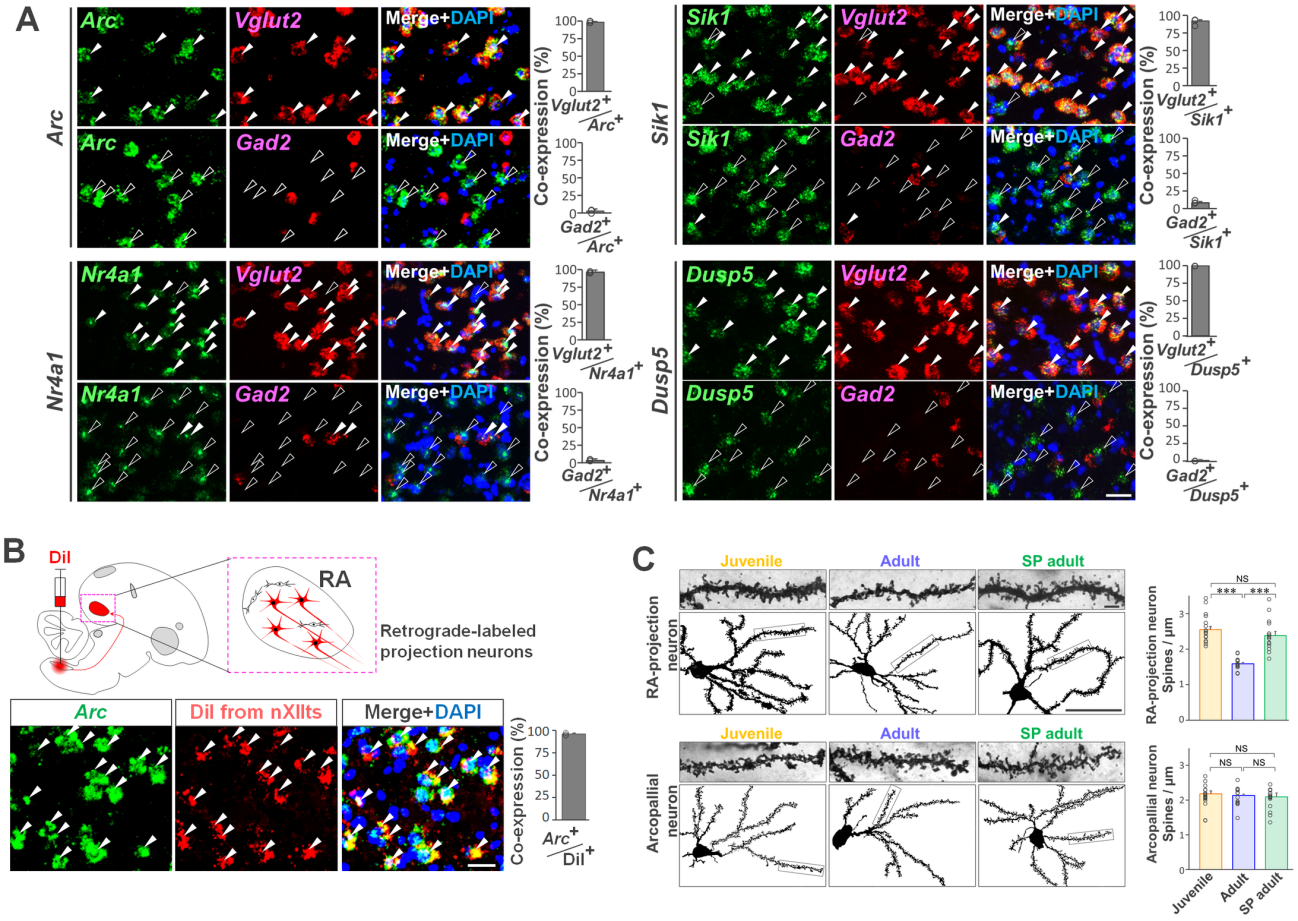
Singing experience–mediated dendritic spine pruning in RA projection neurons. (A) Coinduction of RA cluster I genes (*Arc*, *Nr4a1*, *Sik1*, and *Dusp5*) after juvenile singing in glutamatergic neurons with *Vglut2* (+) but not GABAergic neurons with *Gad2* (+). Filled and empty arrowheads: cells that coexpressed or did not coexpress with singing activity–dependent genes and cell marker genes, respectively. Cell nuclei (blue, DAPI). Scale bar = 40 μm. Bar graphs: proportion of each subpopulation in cells that express the mRNA of RA Cluster I genes. (B) Selective induction of *Arc* mRNA (green) after juvenile singing in RA projection neurons. Diagram of DiI retrograde labeling (red) of RA projection neurons to nXIIts. Cell nuclei (blue, DAPI). (C) Golgi-stained RA projection neurons and RA-surrounding arcopallial neurons in juvenile (55 phd), adult (105 phd), and SP adult (101 phd at 1–2 days after release) birds. Scale bars = 5 μm (upper) and 50 μm (lower). Bar graphs: dendritic spine density of RA projection neurons and RA-surrounding arcopallial neurons in juveniles (*n* = 18 cells from 6 birds), adults (*n* = 15 cells from 5 birds), and SP adults. ***P < 0.0001, NS: *p* > 0, unpaired *t* test with Bonferroni correction. Error bars: SEM. Supporting data can be found in S5 Data. NS, not significant; phd, post hatching day; RA, robust nucleus of the arcopallium; SP, singing-prevented.

Precise and reliable neural activity driven by the functional neural connectivity between excitatory and inhibitory neurons within premotor circuits is critical for the production of structured song patterns [32–35]. We therefore examined which types of RA neuron expressed the cumulative singing experience–regulated genes. By colabeling with a glutamatergic excitatory neuron maker *Vglut2* or a GABAergic inhibitory neuron maker *Gad2* [36], we identified that the tested cumulative singing experience–regulated genes—*Arc*, *Nr4a1*, *Sik1*, and *Dusp5*—were coinduced in the glutamatergic excitatory neurons, not GABAergic interneurons, of RA after singing (Fig 5A). Furthermore, by colabeling with DiI retrograded from nXII, we confirmed neurons expressing the singing experience–regulated genes projected to nXII, innervating vocal and respiratory musculature [37] (Fig 5B).

Dendritic spine density in RA projection neurons was associated with vocal plasticity and reduced through the critical period of song learning [38,39]. Therefore, we measured whether singing experience regulated dendritic spine density in the RA projection neurons. We found that both SP adults and normal juveniles retained a higher density of dendritic spines in the RA projection neurons compared with normal adults (Bonferroni-corrected unpaired *t* test: juvenile:adult, *t*(31) = 9.14, *p* = 2.6e-10; SP adult:adult, *t*(28) = 6.59, *p* = 3.8e-7) (Fig 5C and S7 Fig). In contrast, the arcopallial region surrounding RA, which is a nonvocalization-related area, did not show significant differences in the number of dendritic spines between the 3 groups, supporting the idea that the singing prevention treatment has selective effects on the song-control regions. Although we could identify only a few of the RA interneurons given technical limitation of Golgi staining, these results suggest that cumulative singing experience results in dendritic spine pruning of RA projection neurons that express singing activity–dependent genes.

## Discussion

We revealed here that cumulative singing experience regulates song development, the expression dynamics of activity-dependent genes, and dendritic spine density of RA projection neurons. These results demonstrate that singing practice, rather than age, acts as a nongenetic factor to regulate vocal learning plasticity. A number of neural circuits mature during the critical period of heightened neuronal plasticity early in life [3]. Past studies have shed light on the effects of “passive” sensory experience from the external environment on the regulation of critical periods of sensory systems [40–42]. Sensory input induces gene expression for the functional and structural plasticity of synapses [43,44]. However, the regulatory mechanisms of the critical period of sensorimotor learning for the “active” acquisition of sequential motor skills, such as human language, playing instruments, or birdsong learning, remain unclear. In general, such complex and structured motor patterns do not suddenly emerge through the short-term experience of practice. They rather gradually develop after the cumulative experience of longer periods of self-motivated practice. Therefore, separating the effects of age (intrinsic developmental maturation) and practice (self-motivated behavior) is crucial for precise understanding of the neural mechanisms underlying the critical period of sensorimotor learning.

Compared with birds isolated from sensory learning (tutoring) or auditory feedback during the sensorimotor learning, SP birds produced immature subsong/early plastic songs when they were released from manipulations at adulthood (>100 phd). In contrast to SP birds, both nontutored and auditory feedback–prevented birds can produce singing behavior during the sensorimotor learning phase and develop a certain degree of structured songs by adulthood even without referencing tutor song memories [45–47]. When nontutored birds in social isolation first hear tutor songs at 120 phd, they thereafter change a few syllables of their own already existing song to mimic those from their tutor’s song. However, they do not retain the vocal plasticity necessary to mimic their tutor’s song structures [45,46]. This decreased song plasticity in early isolated birds could be caused by partial closing of sensory and sensorimotor learning ability in nontutored birds at adulthood. In parallel, birds prevented from hearing their own song production through noise exposure can change syllable acoustics but not copy the sequence order of memorized tutor songs when they are released from noise exposure as adults [47]. In contrast to adult birds prevented from matching their song output to memories, SP adult birds retain sufficient vocal learning plasticity to copy both the syllable acoustics and sequence order of memorized tutor songs. This suggests that singing practice itself, rather than other age-related factors, regulates the critical phase of sensorimotor learning. The high degree of vocal learning plasticity in SP birds was associated with producing structurally immature songs when they are allowed to sing freely. Therefore, although interruption of normal tutoring and hearing experiences can retain a certain degree of vocal plasticity until the adult stage, preventing cumulative singing experience induces a more intensive effect on vocal learning plasticity by almost stopping song maturation. This song immaturity could be a direct cause of the later vocal learning plasticity, and it enabled age-independent vocal learning after the sensitive period in the SP birds. However, because of the limited sample size in this study, we could not fully examine whether the amount of singing practice during the prevention period affects individual’s song imitation accuracy after release.

We previously reported that early-deafened zebra finches produce a normal amount of singing during development and still maintain highly variable songs in terms of both syllable acoustics and sequence even as adults (140–180 phd) [20]. Therefore, cumulative singing experience itself is not sufficient to decrease vocal plasticity. Rather, the cumulative experience of sensorimotor integration—i.e., self-motivated vocalization with normal auditory feedback (tutoring)—is an essential behavioral factor to promote song stabilization—i.e., closing of the sensorimotor learning period. However, this study does not examine a potential contribution of cumulative singing experience to closing the sensory learning phase versus the sensorimotor learning phase. To elucidate this point, it would be necessary to track the song development of SP birds that are exposed to a tutor only in adulthood. In addition, singing prevention longer than the 100 days used in the present study would allow us to examine the time limitation of retaining sensorimotor learning plasticity, which might be genetically constrained.

RA has a crucial role in the song circuits as the telencephalic output nucleus receiving 2 premotor inputs: a motor exploration signal from the LMAN as the cortical-basal ganglia-thalamic circuit output and a time-locked sequence signal from HVC of the motor circuit (Fig 2A) [31,48]. However, the 2 circuits do not equally contribute to song production during the critical period of sensorimotor learning. Early in song development, vocal output is dominated by LMAN input to RA [49]. With accumulation of singing experience, the LMAN’s effectiveness is curtailed by pruning and strengthening of the HVC to RA synapses [50,51]. In addition, synaptic connectivity from interneurons to projection neurons in RA is initially dense and nonspecific and then pruned with specific reciprocal patterns during sensorimotor learning [33], suggesting that song development engages multiple processes to reduce shared synaptic inputs to RA projection neurons. If so, our finding that the SP birds retained higher density of dendritic spines in the RA projection neurons like juveniles may indicate a singing experience—but not age-dependent mechanism for sculpting of functional connectivity of RA. Activity-dependent synaptic potentiation and depression are induced at the synapses of RA projection neurons [52]. Notably, zebra finch juveniles (45–60 phd) possess distinct capacity for long-term depression (LTD) in RA neurons compared with adult birds [53]. *Arc* is a critical regulator that modulates the synaptic plasticity underlying LTD induction [54,55]. This evidence supports a functional linkage between activity-dependent synaptic plasticity and activity-dependent gene induction in RA for regulation of vocal plasticity through the singing experience–dependent sculpting of functional connectivity.

Most of the singing experience–regulated genes were previously known as immediate-early genes, which regulate downstream effector proteins for activity-dependent synaptic plasticity [11,56–58]. Singing behavior generates robust neuronal activity in the song system, including RA and other song nuclei, throughout a bird’s life from juvenile to adult stages [31,48,59–61]. However, some immediate-early genes are more highly induced by singing in the song nuclei (e.g., *egr1*[*zenk*] and *Arc* in RA and *penk* in HVC) in juvenile than adult stages, although the causal reason of the developmental different induction is not known [13,17–19]. In this study, we found that RA shows a reduction of singing activity–dependent gene induction during the accumulation of singing experience. Which region-selective neuronal mechanisms regulate change in the expression dynamics of singing activity–dependent genes in RA? One possibility is neuronal activity–mediated epigenetic regulation—i.e., induction and activation of epigenetic regulators by neuronal firing to change epigenetic states at the regulatory regions of other activity-dependent genes [62]. In the RA Cluster I genes, we identified the epigenetic regulators that were induced by singing: a DNA methylation regulator *Gadd45b* [63,64], replacement histone *H3*.*3b* [65], and a subunit of the Sin3–histone deacetylase 1 (HDAC1) complex *Fam60a* [66,67]. These singing-driven epigenetic regulators could directly change the epigenetic state of the regulatory regions of other RA Cluster I genes, but this hypothesis remains to be fully evaluated.

Vocal learning has evolved convergently in a few lineages of birds and mammals. Recently, there is accumulating evidence that marmoset monkeys possess the ability for production-related vocal plasticity, especially to develop acoustic features via feedback from their parents [68–70]. Production-related vocal plasticity in marmosets could be a good mammalian model system to examine the cumulative vocal practice–related expression dynamics of activity-dependent genes that we found in songbirds. Avian vocal learners (songbirds, parrots, and hummingbirds) and humans possess analogous neural networks connecting anatomically similar cortical and subcortical brain regions as a form of convergent neural circuit evolution [16]. In addition, songbirds and humans share convergent transcriptional specializations in the brain regions for learned vocalization [71]. Therefore, language acquisition in humans could be mediated by the expression dynamics of neuronal activity–dependent genes in specific neural populations, such as cortical layer V projection neurons in laryngeal motor regions, analogous to songbirds’ RA [71]. Although many challenges remain in elucidating the neural basis of vocal learning, insights from songbirds may lead to a better understanding of the molecular mechanisms underlying learned vocal communication.

## Materials and methods

### Ethics statement

All experiments were conducted under the guidelines and approval of the Committee on Animal Experiments of Hokkaido University (Approved No. 13–0061). These guidelines are based on the national regulations for animal welfare in Japan (Law for the Humane Treatment and Management of Animals with partial amendment No. 105, 2011). For brain sampling, the birds were humanely killed by decapitation after overdose pentobarbital injection.

### Animals

Zebra finch adult males were obtained from our breeding colonies at Hokkaido University. Birds were kept in breeding cages under a 13:11 hour light/dark cycle. During song-recording sessions, each bird was individually housed in a cage inside a sound-attenuating box.

For vocal practice restriction, singing prevention was performed from the initiation of first singing (at around 30 phd) until adult stage (*n* = 24 birds, 91–133 phd, mean ± SD = 101.6 ± 8.8 phd). During light-on time, juveniles were prevented from singing by a custom-made weight on the neck that shifted their posture slightly toward an inferior position (approximately at 0.5–1.5 cm lower than normal height). The weight was detachable and daily adjusted for each bird (up to 16.5–24.0 g). Note that weight was usually supported by the floor, not carried by the bird’s neck, during the days. Therefore, the birds could freely generate daily behaviors, such as drinking, eating, grooming, and calling. The weight was removed from birds during light-off time and for at least 1 hour during light-on time to reduce potential stress. During the weight-free time each day, the real-time singing behaviors were monitored with Sound Analysis Pro (SAP) and interrupted by light tapping or by opening the sound-attenuating box. No singing behavior was observed during light-off time. Body weight was carefully monitored every 2–3 days. For hearing experience to a tutor song, birds were kept with their biological fathers after hatching until 30 phd and subsequently exposed to their father every 2–4 days until adult stage.

### Song recording and analysis

Songs were recorded using a unidirectional microphone (SM57, Shure) connected to a computer with SAP (v1.04) [72]. Singing duration was defined as the total amount of singing during the last 30 minutes before euthanasia for brain sampling. A song bout was defined as the continuous production of syllables followed by at least 200 ms of silence.

Song motif consistency was measured as the motif similarity score within each day and calculated with the default setting in the SAP software using “time-course” and “symmetric” comparison modes. We randomly selected 20 bouts of songs produced after 3 PM in 1 day. The similarity scores between any 2 of 20 bouts were compared by the round-robin comparison—i.e., a total of 190 similarity scores were calculated at each developmental stage. The similarity score, which represents a global measure of percent similarity, was calculated by comparing syllable acoustic features (e.g., pitch, FM, AM, Wiener entropy, and goodness of pitch) within 9 ms sliding time windows. *P* values for comparisons of motif consistency between developmental stages or conditions were obtained using an unpaired *t* test for different conditions and a paired *t* test among similar conditions with Bonferroni correction.

For the motif-based song similarity analysis, 20 bouts of songs were randomly selected and analyzed at each developmental time point. Song similarity scores were calculated by whole-motif comparison against each pupil’s tutor songs using the of the SAP software. For the syllable-based song similarity analysis, we used 50 syllables from the same song bouts, which were analyzed for the motif-based song similarity. Introductory notes in a song were not included for analyses. The series of separated syllable files of songs were transferred to the CORRELATOR program of Avisoft SASLab pro (Avisoft Bioacoustics, Berlin, Germany) for calculating the similarity scores between the syllables from pupils’ and tutors’ songs by the round-robin comparison [73]. The highest similarity score for each syllable of pupil songs against tutor syllables was averaged as the similarity score of total syllables for each individual.

### Brain sampling

Male zebra finch juveniles (*n* = total 29, 40–55 phd), adults (*n* = total 26, 101–338 phd), and SP adults (*n* = total 12, 91–133 phd at 1–2 days after release from singing prevention) were used for Quartz RNA-seq and in situ hybridization. Each bird was individually placed in a sound-attenuating box overnight, and singing behavior (undirected singing) was recorded during the next morning after lights on. Similarly to above, for brain sampling of silent conditions, birds were prevented from singing (but allowed to produce calls) by light tapping on cages when the birds started singing after lights on. After each session of singing behavior observation, the birds were humanely killed by decapitation. Brains were embedded in OCT compound (Sakura Fine Technical) and stored at −80 °C until use.

### Quartz RNA-seq

For sampling of song nuclei and RNA extraction, male zebra finch juveniles after 45 minutes silent (*n* = 3, 47–48 phd), juveniles with 45 minutes singing (*n* = 3, 40–50 phd), adults with 45 minutes silent (*n* = 2, 101–104 phd), adults with 45 minutes singing (*n* = 4, 110–338 phd), and SP adults with 45 minutes singing (*n* = 3, 96–101 phd at 1–2 days after release from singing prevention) were used (S1 Table). For identification of clear RA and HVC boundaries against surrounding nonvocal areas under microscope observation, a fluorescent-retrograde tracer, Cholera Toxin B subunit conjugated with AlexaFluor555 (Invitrogen, 1 mg/μl in 1× PBS, 100 nl/hemisphere), was injected into RA 10 days before euthanasia. After behavioral observations, birds were decapitated, and brains were removed and stored at −80°C until sectioning. Brain sections were cut at a 20 μm thickness in the sagittal plane and mounted onto glass slides with a handmade membrane system for laser microdissection. Fluorescent-labeled RA and HVC tissues were microdissected from 14 to 20 brain slices using a laser capture microscope ArcturusXT (Arcturus Bioscience). The collected tissue was dissolved in Qiagen RLT buffer. Total RNA was purified using a column-based method (RNeasy Micro kit; Qiagen) and treated with DNase in the column to avoid contamination of genomic DNA. RNA integrity number (RIN) and concentration were measured with a Bioanalyzer2100 (Agilent Technologies) to confirm RNA quality (RIN: 6.4–7.4, RNA concentration: 3.5–10 ng/μl).

For cDNA synthesis, amplification, and library preparation, cDNA was amplified from purified total RNA using previously reported methods [74]. Total RNA (10 ng) was used for synthesis of first-strand cDNA. The following PCR amplification was performed with 14 PCR cycles at 98 °C for 10 seconds, 65 °C for 15 seconds, and 68 °C for 5 minutes. The amplified cDNA samples were purified using a PCR purification column (MiniElute PCR Purification Kit; Qiagen). To check the quality of amplified cDNA, concentrations and smearing patterns of cDNA samples were measured with a Bioanalyzer 2100 (cDNA amount, 72–434 ng) (S1 Table). Amplified cDNA samples (10 ng) were fragmented to 100–300 bp in size using a DNA Shearing System LE220 (peak incident power 450 W, duty factor 30%, cycle/burst 200, and treatment time of 700 seconds) (Covaris) and then purified by a Zymo DNA 5 column. After end repair of DNA fragments, adaptors were ligated and amplified using a ligation-based Illumina multiplex library preparation method (LIMprep) with a KAPA Hyper Prep Kit (Nippon genetics) and 7 PCR cycles. All libraries were then sequenced using a Hiseq 2500 Sequencer (Illumina) for 100 bp single-end sequencing. Library preparation was performed in the Bioinformatics Research Unit at RIKEN Advanced Center for Computing and Communication under supervision by Drs. Y. Sasagawa and A. Nikaido. All Quartz RNA-seq data from zebra finches were deposited in the DDBJ Sequence Read Archive (submission number DRA005559).

### Improvement of gene annotation file of zebra finch brain transcripts

The previous gene annotation file from Ensemble (*Taeniopygia_guttata* taeGut3.2.4.76.gtf) did not include 3′ UTR information. For annotation of read sequences obtained from the RNA-seq data, the lack of 3′ UTR information decreases the chances of accurate estimations of gene expression. Therefore, we elongated the annotation information with our RNA-seq data from zebra finch whole-brain samples (S1 Table). Total RNA was isolated from the pallium and pallidum regions of adult male zebra finches under silent and dark conditions (*n* = 5, 234–786 phd) using TRIzol Reagent (Invitrogen) according to manufacturer’s protocol (Invitrogen) and then column purified using a RNeasy Micro kit (Qiagen). Samples were treated with RNase-free DNase. The total RNA samples were used for library synthesis with TruSeq DNA Sample Prep Kits (Illumina). All libraries were then sequenced using the Illumina Hiseq 2500 Sequencer for 100 bp paired ends. These experimental steps were performed in Dr. Y. Suzuki’s laboratory in the Department of Computational Biology at the University of Tokyo. The 33.5–47.0 M reads for each telencephalon brain sample were output from the Illumina Hiseq 2500. Sequencing reads were mapped onto the ZF reference genome obtained from Ensemble (*Taeniopygia_guttata* taeGut3.2.4.dna.fa) with the Tophat2 program and assembled to predicted transcripts with the Cufflinks program. By comparison with the previous annotation file using the cuffcompare program, 12,156 transcripts were identified as predicted RNA transcripts expressed in the zebra finch telencephalon. The RNA-seq data from the zebra finch telencephalon were deposited in the DDBJ Sequence Read Archive (submission number DRA005548 and DRA005559).

### Identification of differentially expressed genes in HVC and RA

Total RNA-seq reads (9.7–20.9 M) from zebra finch juveniles after 45 minutes silent, juveniles with 45 minutes singing, adults after 45 minutes silent, and adults with 45 minutes singing were used. First, RNA-seq reads were mapped onto the zebra finch reference genome with the Tophat2 program, and then the fragments per kilobase of exon per million mapped fragments (FPKM) of each transcript (12,156 genes) was calculated using the Cufflinks program. Principal component analysis (PCA) using the prcomp package in R was used to check whether there were outliers of quality of RNA-seq. A Bonferroni-corrected DEseq2 was used to identify the differentially expressed genes between juveniles and adults (*P* < 0.05; 1,540 genes in HVC and 1,352 genes in RA), singing and silent conditions (*P* < 0.05; 385 genes in HVC and 266 genes in RA), and juvenile singing and adult singing conditions (*P* < 0.05; 937 genes in RA and 2,443 genes in HVC) in normal birds. A total of 3,214 genes in HVC and 1,811 genes in RA were detected as differentially expressed genes.

### WGCNA

WGCNA is a biologically meaningful technique for quantifying similarity of expression patterns among all pairs of probes across all treatment conditions [75,76]. WGCNA identifies modules of densely interconnected probes by hierarchical clustering based on topological overlap and by assigning each probe to a “Cluster (module)” based on shared expression patterns. A WGCNA was performed using the WGCNA R package to further investigate the relationship between biological traits (singing experience, age, and/or singing induction) and identify coregulated gene clusters. General information about network analysis methodology and WGCNA software is available at http://labs.genetics.ucla.edu/horvath/htdocs/CoexpressionNetwork/. Pairwise Pearson correlation coefficients were calculated for all detected genes. The resulting Pearson correlation matrix was transformed into a matrix of connection strength (an adjacency matrix) using the power function [(1 + correlation) / 2 × soft threshold power], which was then converted to a topological overlap matrix. A preliminary network was built to assess overall connectivity. From this network, 3,214 and 1,811genes in HVC and RA, respectively, with the highest connectivity were retained for subsequent WGCNA (soft threshold power = 10, corType = pearson, minModuleSize = 30, detectCutHeight = 0.98, and merge CutHeight = 0.4). Clusters were defined as branches of the dendrogram obtained from clustering and were labeled with colors beneath the dendrograms. To study the relationship between expression variability within the clusters and behavioral trait variability, correlations were computed between the principal components of each module and traits. *P* values were computed for each correlation.

To further confirm the WGCNA results, we separately compared the expression profiles of the differentially expressed genes (1,811 genes) in RA under singing condition. DESeq2 (*P* < 0.05) was performed to compare the FPKM values of each transcript between adult singing (*n* = 4) and SP adult singing (*n* = 3) and between juvenile singing (*n* = 3) and SP adult singing (*n* = 3). We then counted the overlap between the Cluster I gene and genes detected by DESeq2.

### *Cis*-enrichment analysis

Information on transcription factors (TFs) and their binding motifs was obtained from the JASPAR 2018 database (http://jaspar.genereg.net, CORE Vertebrata). We then used 349 genes in the CIS-BP database as TFs existing in the zebra finch genome (*T*. *guttata* taeGut3.2.4.dna.fa). The upstream 1 kb sequences of the TSSs of the 12,156 genes that were expressed in the telencephalon were extracted from the zebra finch genomic sequence. A total of 7,052 genes, including 61 and 519 genes in RA Gene Cluster I and II, respectively, were used for further *cis*-enrichment analysis, because the other 5,104 genes possessed a sequence gap (nonsequenced region) in their upstream 1 kb from TSS.

The Find Individual Motif Occurrences (FIMO) program was used to search for TF binding sites in the upstream 1 kb of the TSS (*p* < 10^4^, for each binding site). For each TF, we calculated how many RA Gene Cluster I and II genes had the TF binding sites in the upstream 1 kb. To predict TFs of the cluster genes, the frequency appearance of binding sites was compared with the background frequency of 7,052 genes for each TF (both Fisher’s test and G test, *p* < 0.05).

### In situ hybridization

cDNA fragments used for the synthesis of in situ hybridization probes were cloned from a whole-brain cDNA mixture of a male zebra finch. Total RNA was transcribed to cDNA using Superscript Reverse Transcriptase (Invitrogen) with oligo dT primers. The cDNAs were amplified by PCR using oligo DNA primers directed to conserved regions of the coding sequence from the NCBI cDNA database (S2 Table). PCR products were ligated into the pGEM-T Easy plasmid (Promega). The cloned sequences were searched using NCBI BLAST/BLASTX to compare with homologous genes to other species and identified genome loci using BLAT of UCSC Genome Browser.

For radioisotope in situ hybridization, male zebra finch juveniles (*n* = total 23, 45–55 phd), adults (*n* = total 20, 103–227 phd), and SP adults (*n* = total 12, 91–133 phd at 1–2 days after release from singing prevention) were split into 6 experimental groups: (I) juvenile 30 minutes silent, (II) adult 30 minutes silent, (III) juvenile 30 minutes singing, (IV) adult 30 minutes singing, (V) SP adults with 30 minutes silent, and (VI) SP adults with 30 minutes singing. Frozen sections (12 μm thick) were cut in the sagittal plane. Brain sections for a given experiment were simultaneously fixed in 3% paraformaldehyde/1× PBS (pH 7.4), washed in 1× PBS, acetylated, dehydrated in an ascending ethanol series, air dried, and processed for in situ hybridization with antisense ^35^S-UTP-labeled riboprobes of genes. To generate the riboprobes, gene inserts in the pGEM-T Easy vector were PCR amplified with plasmid M13 forward and reverse primers and then gel purified. The amplified DNA fragments and SP6 or T7 RNA polymerase was used to transcribe the antisense ^35^S-riboprobes. A total of 1 × 10^6^ cpm of the ^35^S-probe was added to a hybridization solution (50% formamide, 10% dextran, 1× Denhardt’s solution, 12 mM EDTA [pH 8.0], 10 mM Tris-HCl [pH 8.0], 300 mM NaCl, 0.5 mg/mL yeast tRNA, and 10 mM dithiothreitol). Hybridization was performed at 65 °C for 12–14 h. The slides were washed in 2× SSPE and 0.1% β-mercaptoethanol at RT for 1 h, 2× SSPE, 50% formamide, and 0.1% β-mercaptoethanol at 65 °C for 1 h, and 0.1× SSPE twice at 65 °C for 30 minutes each. Slides were dehydrated in an ascending ethanol series and exposed to X-ray film (Biomax MR, Kodak) for 1–14 days. We carefully attended not to overexpose X-ray films to S^35^-riboprobe hybridized brain sections. The slides were then dipped in an autoradiographic emulsion (NTB2, Kodak), incubated for 1–8 weeks, and processed with D-19 developer (Kodak) and fixer (Kodak). Developed slides were Nissl-stained with a cresyl violet acetate solution (Sigma) for the capture of high-resolution images. For quantification of mRNA signal, exposed X-ray films of brain images were digitally scanned under a microscope (Leica, Z16 APO) connected to a CCD camera (Leica, DFC490) with Application Suite V3 imaging software (Leica), as previously described [12,13,19,20]. To minimize handling bias for signal detection among experimental groups, we performed in situ hybridization using multiple brain sections at once for each probe and exposed S^35^-riboprobe hybridized brain sections on the same sheet of X-ray films. The same light settings were used for all images. Photoshop (Adobe Systems) was used to measure the mean pixel intensities in the brain areas of interest from sections after conversion to 256 grayscale images. For statistical analysis of the expression of each gene, we performed analysis of covariance (ANCOVA) to examine the homoscedasticity from the regression line of the gene induction ratio between singing duration and expression level.

For fluorescence in situ hybridization, dinitrophenyl (DNP)—and digoxigenin (DIG)-labeled riboprobes were used. A total of 100–200 ng of the DNP/DIG-labeled riboprobe was mixed with the hybridization solution (50% formamide, 10% dextran, 1× Denhardt’s solution, 1 mM EDTA [pH 8.0], 33 mM Tris-HCl [pH 8.0], 600 mM NaCl, 0.2 mg/mL yeast tRNA, 80 mM dithiothreitol, and 0.1% N-lauroylsarcosine). Hybridization was performed at 68 °C for 6–13 h. Washing steps were performed as follows: 5× SSC solution at 68 °C for 30 minutes, formamide-I solution (4× SSC, 50% formamide, and 0.005% Tween20) at 68 °C for 40 minutes, formamide-II solution (2× SSC, 50% formamide, and 0.005% Tween20) at 68 °C for 40 minutes, 0.1× SSC 68 °C 15 minutes × 3, NTE buffer at RT for 20 minutes, and TNT buffer × 3, and TNB buffer (0.5% blocking reagent [Perkin Elmer]/1× TNT buffer) at RT for 30 minutes. DNP-labeled probes were detected with an anti-DNP horseradish peroxidase (HRP)-conjugated antibody using a TSA DNP system (Perkin Elmer) and anti-DNP KLH AlexaFluor488 (Molecular Probes, cat#A-11097). Following treatment with 1% H_2_O_2_/1× PBS for 30 minutes, DIG-labeled probes were detected with anti-DIG HRP-conjugated antibody (Jackson Laboratory, cat#200-032-156) and a TSA Plus Cy3 system (Perkin Elmer). Signal images were obtained by fluorescence microscopy (EVOS FL; Thermo Fisher Science).

### Retrograde labeling of RA projection neurons

nXIIts of juvenile birds (30–34 phd) was targeted with stereotaxic coordinates in mm: −0.8 rostral, 0.2 lateral, and 5.9–6.0 ventral from the bifurcation of the central sinus at the border of the forebrain and cerebellum. The retrograde tracer DiI (SIGMA, 70 mg/ml dissolved in N, N-dimethylformamide; 100 nl) was injected into the nXIIts 10 days before euthanasia. Birds were humanely killed after 30 minutes of singing, and brains were processed for fluorescence in situ hybridization.

### Golgi staining

Zebra finch male juveniles (*n* = 6, 46–55 phd), adults (*n* = 5, 106–796 phd), and SP adults (*n* = 5, 100–101 phd) were used for Golgi staining. Brain tissues were sampled under silent and dark conditions and incubated in the impregnation solution from an FD Rapid GolgiStain kit (FD NeuroTechnologies) for 2 weeks in the dark, incubated in a replacement solution for 3 days, embedded in OCT compound (Sakura Fine Technical), and stored at –80 °C until sectioning. Brain sections with a thickness of 100 μm were cut in the sagittal plane. Sections were dried at RT, rinsed with water, and immersed in a staining solution (FD NeuroTechnologies). After staining, sections were dehydrated in an EtOH series, immersed in xylene, and then mounted with Permount. Dendritic spines were counted using photo images taken at 100× magnification with a BZ-X710 Microscope (Keyence). Golgi-stained cell images were obtained by Z-stacking images with 100 planes positioned at a distance of every 0.2 μm. Dendritic spine density was calculated for 3 RA projection neurons and 3 surrounding neurons in the arcopallium for each bird (*n* = 18 neurons from 6 juveniles, *n* = 15 neurons from 5 adults, and *n* = 15 neurons from 5 SP adults). Although only a few of the RA interneurons could be identified by the Golgi staining, RA projection neurons could be distinguished from interneurons by their characteristic mossy dendritic and axonal morphologies (S7B Fig) [36].

### Statistical analysis

Data for song motif consistency were analyzed using an unpaired *t* test for different conditions and a paired *t* test with Bonferroni correction for multiple comparisons (Fig 1E). Data for differentially regulated genes in RNA-seq were obtained using DEseq2 with Bonferroni correction and subsequently analyzed using a WGCNA (Fig 2C–2F). Data for induction of singing activity–regulated genes were analyzed using ANCOVA with Bonferroni correction (Fig 4A and 4B and S3–S5 Figs). Dendritic spine density data were compared using an unpaired *t* test with Bonferroni correction (Fig 5C).

## Supporting information

S1 Fig Singing prevention did not influence body weight and call acoustics. (A) A zebra finch with (right) and without (left) a custom-made weight on the neck for posture manipulation to prevent singing. (B) Body weights of normal juveniles (*n* = 10; 45–51 phd, mean 47.5 ± 2.0 SD), adults (*n* = 10; 102–310 phd, mean 139.5 ± 65.3 SD), and SP adults (*n* = 10; 100–101 phd, mean 100.6 ± 0.7 SD) (NS: *P* > 0.05, 1-way ANOVA with Bonferroni correction). (C) Comparison of the call acoustic features, syllable duration, pitch, pitch goodness, AM, and entropy variance between normal adults and SP adults after being released from signing prevention (NS: *P* > 0.05, Student *t* test). (D) Examples of the songs in 3 persistent singers under singing prevention. Top panels: their tutor songs. Blue lines indicate the motif structure of songs. Colored syllables were learned from the same colored syllables of their tutor songs. Supporting data can be found in S6 Data for panels B and C. AM, amplitude modulation; NS, not significant; phd, post hatching day; SP, singing-prevented.
(TIF)

S2 Fig Song development of SP birds after release from singing prevention at adulthood. (A) Song development of SP birds after being released from singing prevention at adulthood. Top panels: their tutor songs. Blue lines indicate the motif structure of songs. Colored syllables were learned from the same colored syllables of their tutor songs. (B) Development of motif consistency of song in normal (red, *n* = 5) and SP (green, *n* = 5) birds. Error bar: SD. (C, D) Development of syllable and motif similarities of songs in SP birds after release from singing prevention (*n* = 5). Error bar: SD. Supporting data can be found in S6 Data for panels B–D. SP, singing-prevented.
(TIF)

S3 Fig Expression heat maps of RA cluster III and IV genes. Heat maps of Z scores of RA Cluster III and IV genes (793 and 63 genes, respectively) normalized by the average expression value of each gene at the juvenile silent condition. Supporting data can be found in S6 Data. RA, robust nucleus of the arcopallium.
(TIF)

S4 Fig Singing prevention extends juvenile-like *Arc* induction until adulthood. (A) Expression of *Arc* mRNA in juveniles (silent, 52 phd; singing, 47 phd), adults (silent, 124 phd; singing, 112 phd), and SP adults just after release from singing prevention (silent, 100 phd; singing, 100 phd). Singing duration (s) is shown at the bottom. White color: *Arc* mRNA expression. Red color: cresyl violet counter stain. Scale bar = 1.5 mm. (B) Higher magnification images showing *Arc* mRNA expression in the song nuclei. Scale bar = 200 μm. (C) Expression dynamics of *Arc* mRNA after singing in the song nuclei (HVC, RA, NIF, LMAN, Area X, and DLM) in juveniles (*n* = 17), adults (*n* = 17), and SP adults at 1–2 days after release from prevention (*n* = 11). Lines represent the linear approximation curve. **P < 0.001, ****P < 0.00001, ANCOVA with Bonferroni correction. Supporting data can be found in S4 Data. A, arcopallium; ANCOVA, analysis of covariance; DLM, dorsal lateral nucleus of the medial thalamus; Hp, hippocampus; LMAN, lateral magnocellular nucleus of the anterior nidopallium; M, mesopallium; N, nidopallium; NIF, interfacial nucleus of the nidopallium; RA, robust nucleus of the arcopallium; SP, singing-prevented; St, striatum; Th, thalamus.
(TIF)

S5 Fig Expression dynamics of singing activity–dependent genes in RA Gene Cluster I in the song nuclei. Expression patterns of *Crem*, *Nr4a1*, *Sik*1, *Fam60a*, *Dusp5*, *Dusp6*, *Atf3*, *Odc*, *c-fos*, *Egr1*, *H3*.*3b*, and *Gadd45β* in silent and singing conditions of juveniles, adults, and SP adults 1–2 days after release from singing prevention. Expression dynamics of the genes after singing in the song nuclei (HVC, RA, NIF, LMAN, Area X, and DLM) in juveniles (orange), adults (purple), and SP adults (green). Lines represent the linear approximation curve. (*p < 0.01, **p < 0.001, ***p < 0.0001, ****p < 0.00001; ANCOVA with Bonferroni correction). Supporting data can be found in S4 Data. ANCOVA, analysis of covariance; DLM, dorsal lateral nucleus of the medial thalamus; LMAN, lateral magnocellular nucleus of the anterior nidopallium; NIF, interfacial nucleus of the nidopallium; RA, robust nucleus of the arcopallium; SP, singing-prevented.
(TIF)

S6 Fig Expression of age-regulated genes in RA Gene Cluster II in the song nuclei. Expression patterns of *Gabra5*, *Evl*, *Dpysl3*, and *Il1rapl2* from RA Cluster II genes under silent and singing conditions in juveniles, adults, and SP adults 1–2 days after release from singing prevention. Expression dynamics of the genes in the song nuclei (HVC, RA, NIF, LMAN, Area X, and DLM) in juveniles (orange), adults (purple), and SP adults (green). Lines represent the linear approximation curve. (*p < 0.01, **p < 0.001, ***p < 0.0001, and ****p < 0.00001; ANCOVA with Bonferroni correction). Supporting data can be found in S4 Data. ANCOVA, analysis of covariance; DLM, dorsal lateral nucleus of the medial thalamus; LMAN, lateral magnocellular nucleus of the anterior nidopallium; NIF, interfacial nucleus of the nidopallium; RA, robust nucleus of the arcopallium; SP, singing-prevented.
(TIF)

S7 Fig Developmental changes in the dendritic spine density of RA projection neurons and examples of Golgi-stained RA interneurons. (A) Developmental changes in the dendritic spine density of RA projection neurons in juveniles (*n* = 6: orange), adults (*n* = 5: purple), and SP adults at 1–2 days after release from singing prevention (*n* = 5: green). Error bars: SD. Supporting data can be found in S6 Data. (B) Golgi-stained RA interneurons in normal adult (left) and SP (right) birds. Scale bars = 5 μm (upper) and 50 μm (lower). RA, robust nucleus of the arcopallium; SP, singing-prevented.
(TIF)

S1 Table Information of brain sampling condition and RNA-seq. RNA-seq, RNA sequencing.
(TIF)

S2 Table PCR primers for cloning of cDNA probes for in situ hybridization. (TIF)

S1 Data Underlying data for Fig 1A, 1B, 1C, 1D, 1E and 1F. (XLSX)

S2 Data Underlying data for Fig 2C, 2D, 2E, 2F and 2G. (XLSX)

S3 Data Underlying data for Fig 3A and 3B. (XLSX)

S4 Data Underlying data for Fig 4A and 4B and S4–S6 Figs. (XLSX)

S5 Data Underlying data for Fig 5A, 5B and 5C. (XLSX)

S6 Data Underlying data for S1B, S1C, S2B, S2C, S2D, S3 and S7 Figs. (XLSX)

## Abbreviations

AFP: anterior forebrain pathway
AM: amplitude modulation
ANCOVA: analysis of covariance
DIG: digoxigenin
DLM: dorsal lateral nucleus of the medial thalamus
DNP: dinitrophenyl
FIMO: Find Individual Motif Occurrences
FPKM: fragments per kilobase of exon per million mapped fragments
HRP: horseradish peroxidase
LMAN: lateral magnocellular nucleus of the anterior nidopallium
LTD: long-term depression
NIf: interfacial nucleus of the nidopallium
NS: not significant
PCA: principal component analysis
phd: post-hatching day
RA: robust nucleus of the arcopallium
RIN: RNA integrity number
SAP: Sound Analysis Pro
SP: singing-prevented
TF: transcription factor
TSS: transcription start site
VMP: vocal motor pathway
WGCNA: weighted gene coexpression network analysis
