# Supplementary figures and images for: Vocal practice regulates singing activity–dependent genes underlying age-independent vocal learning in songbirds
Source: PLoS Biol. 2018 Sep 12;16(9):e2006537. doi: 10.1371/journal.pbio.2006537 (PMC6152990; doi:10.1371/journal.pbio.2006537)

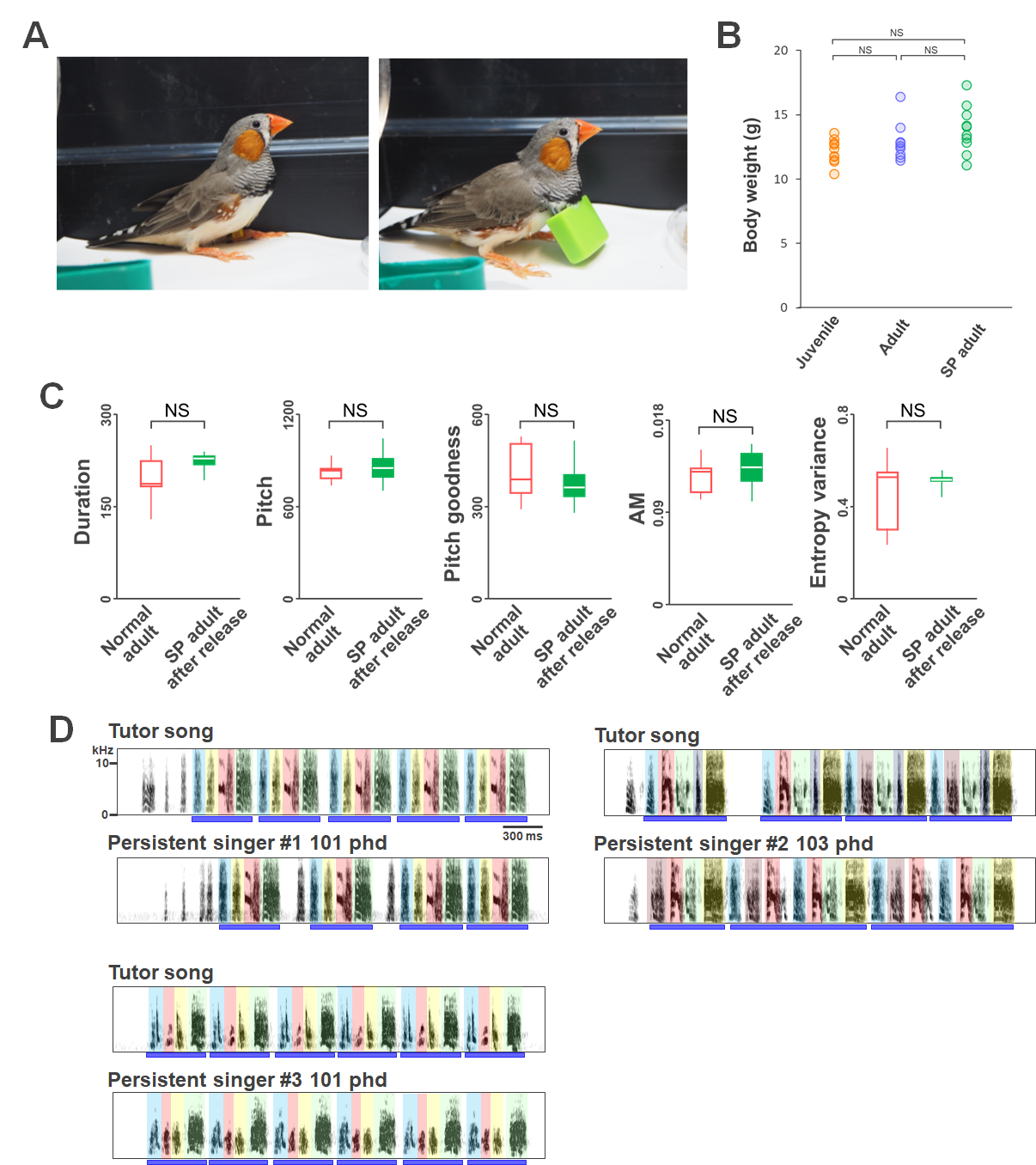

Supplement: S1 Fig — (A) A zebra finch with (right) and without (left) a custom-made weight on the neck for posture manipulation to prevent singing. (B) Body weights of normal juveniles (n = 10; 45–51 phd, mean 47.5 ± 2.0 SD), adults (n = 10; 102–310 phd, mean 139.5 ± 65.3 SD), and SP adults (n = 10; 100–101 phd, mean 100.6 ± 0.7 SD) (NS: P > 0.05, 1-way ANOVA with Bonferroni correction). (C) Comparison of the call acoustic features, syllable duration, pitch, pitch goodness, AM, and entropy variance between normal adults and SP adults after being released from signing prevention (NS: P > 0.05, Student t test). (D) Examples of the songs in 3 persistent singers under singing prevention. Top panels: their tutor songs. Blue lines indicate the motif structure of songs. Colored syllables were learned from the same colored syllables of their tutor songs. Supporting data can be found in S6 Data for panels B and C. AM, amplitude modulation; NS, not significant; phd, post hatching day; SP, singing-prevented. (TIF) [file pbio.2006537.s001.tif]

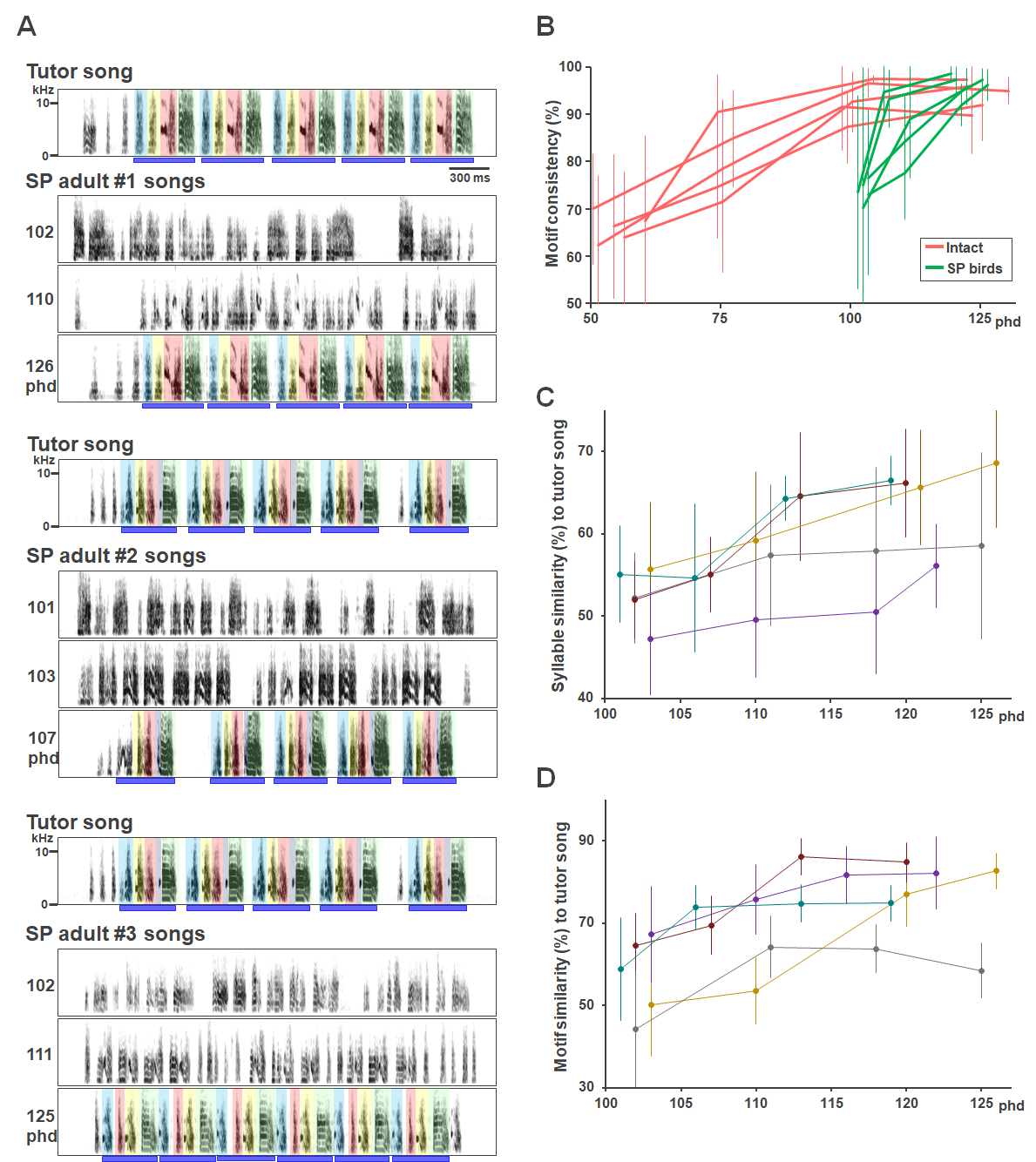

Supplement: S2 Fig — (A) Song development of SP birds after being released from singing prevention at adulthood. Top panels: their tutor songs. Blue lines indicate the motif structure of songs. Colored syllables were learned from the same colored syllables of their tutor songs. (B) Development of motif consistency of song in normal (red, n = 5) and SP (green, n = 5) birds. Error bar: SD. (C, D) Development of syllable and motif similarities of songs in SP birds after release from singing prevention (n = 5). Error bar: SD. Supporting data can be found in S6 Data for panels B–D. SP, singing-prevented. (TIF) [file pbio.2006537.s002.tif]

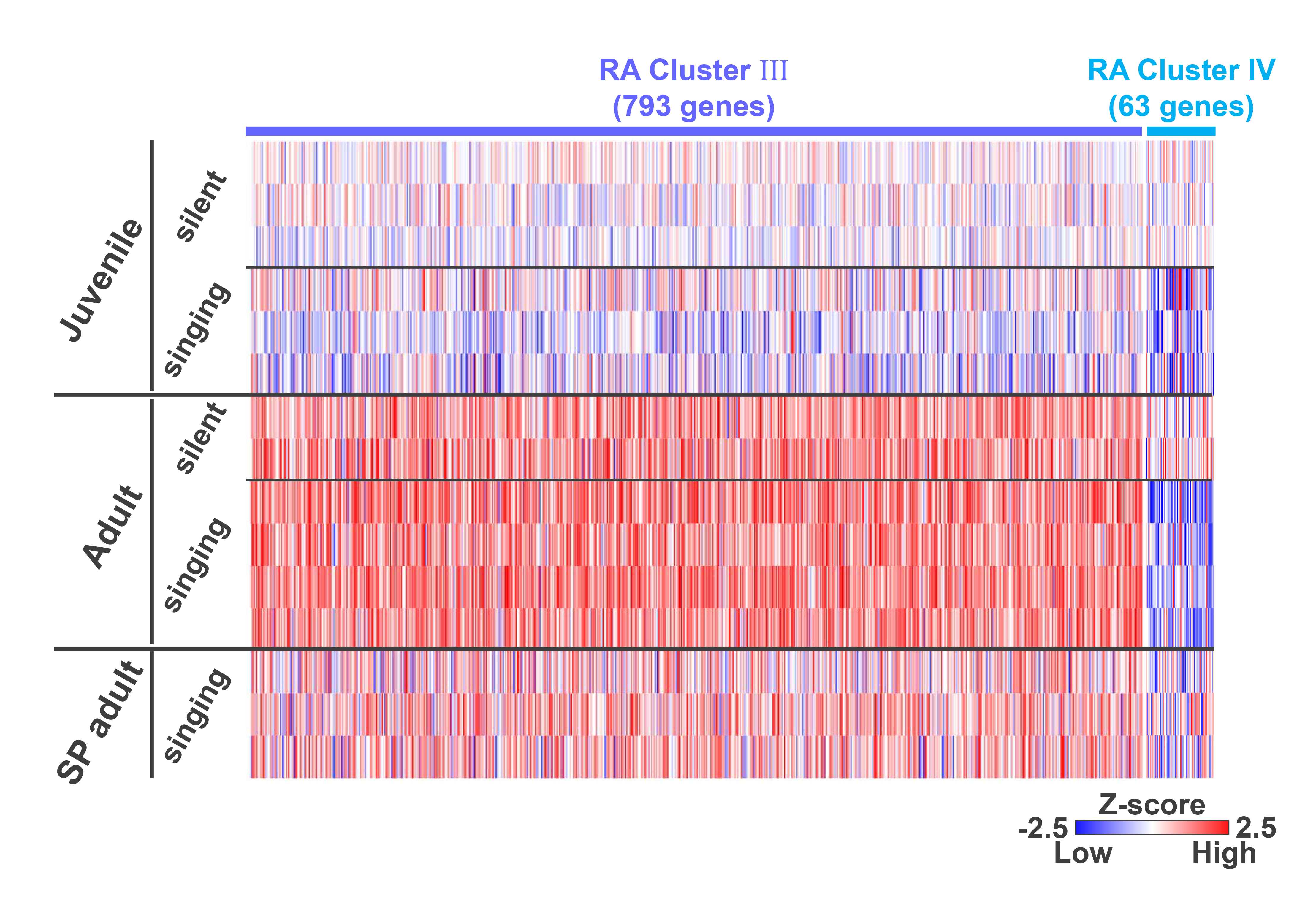

Supplement: S3 Fig — Heat maps of Z scores of RA Cluster III and IV genes (793 and 63 genes, respectively) normalized by the average expression value of each gene at the juvenile silent condition. Supporting data can be found in S6 Data. RA, robust nucleus of the arcopallium. (TIF) [file pbio.2006537.s003.tif]

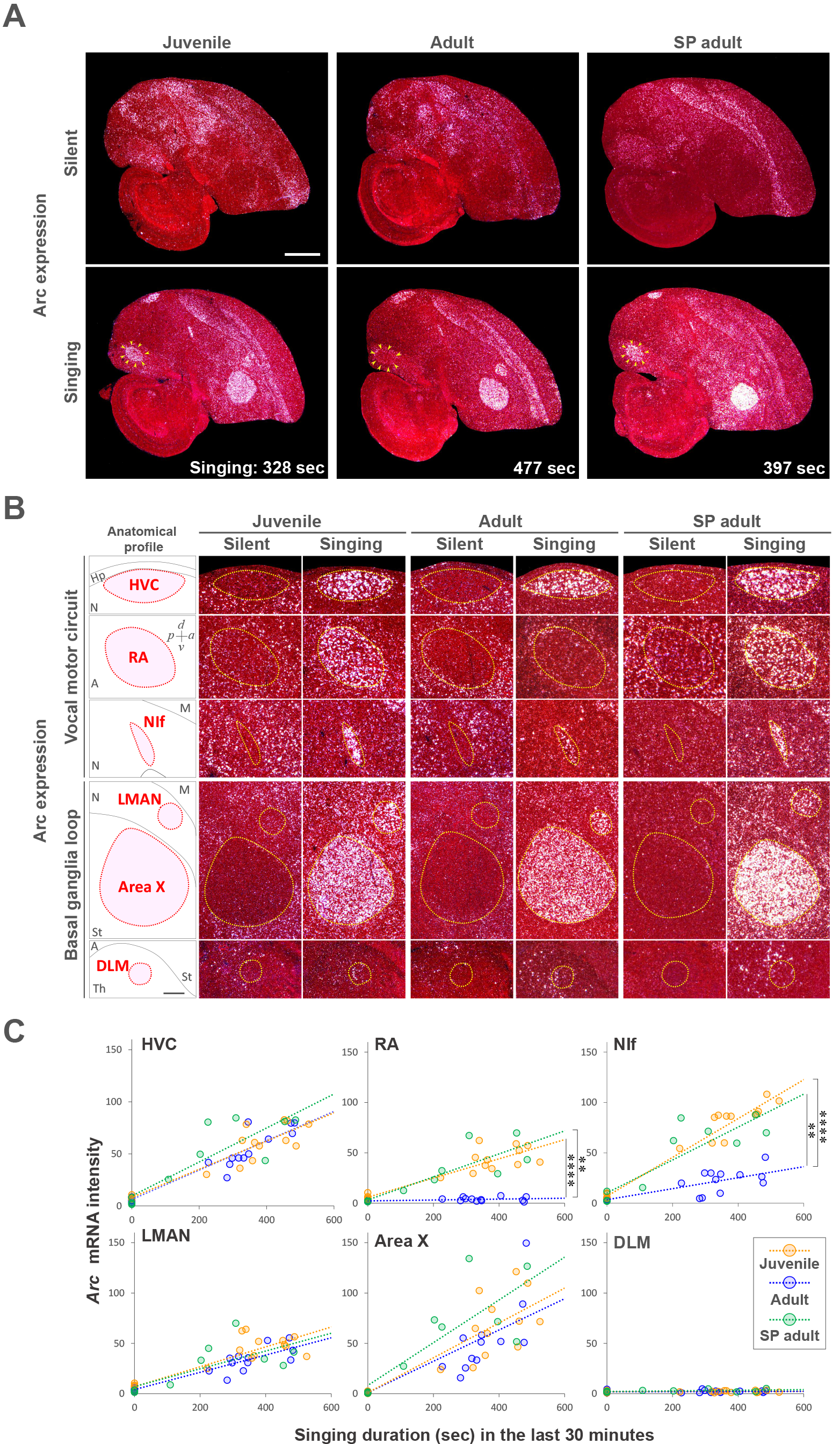

Supplement: S4 Fig — (A) Expression of Arc mRNA in juveniles (silent, 52 phd; singing, 47 phd), adults (silent, 124 phd; singing, 112 phd), and SP adults just after release from singing prevention (silent, 100 phd; singing, 100 phd). Singing duration (s) is shown at the bottom. White color: Arc mRNA expression. Red color: cresyl violet counter stain. Scale bar = 1.5 mm. (B) Higher magnification images showing Arc mRNA expression in the song nuclei. Scale bar = 200 μm. (C) Expression dynamics of Arc mRNA after singing in the song nuclei (HVC, RA, NIF, LMAN, Area X, and DLM) in juveniles (n = 17), adults (n = 17), and SP adults at 1–2 days after release from prevention (n = 11). Lines represent the linear approximation curve. **P < 0.001, ****P < 0.00001, ANCOVA with Bonferroni correction. Supporting data can be found in S4 Data. A, arcopallium; ANCOVA, analysis of covariance; DLM, dorsal lateral nucleus of the medial thalamus; Hp, hippocampus; LMAN, lateral magnocellular nucleus of the anterior nidopallium; M, mesopallium; N, nidopallium; NIF, interfacial nucleus of the nidopallium; RA, robust nucleus of the arcopallium; SP, singing-prevented; St, striatum; Th, thalamus. (TIF) [file pbio.2006537.s004.tif]

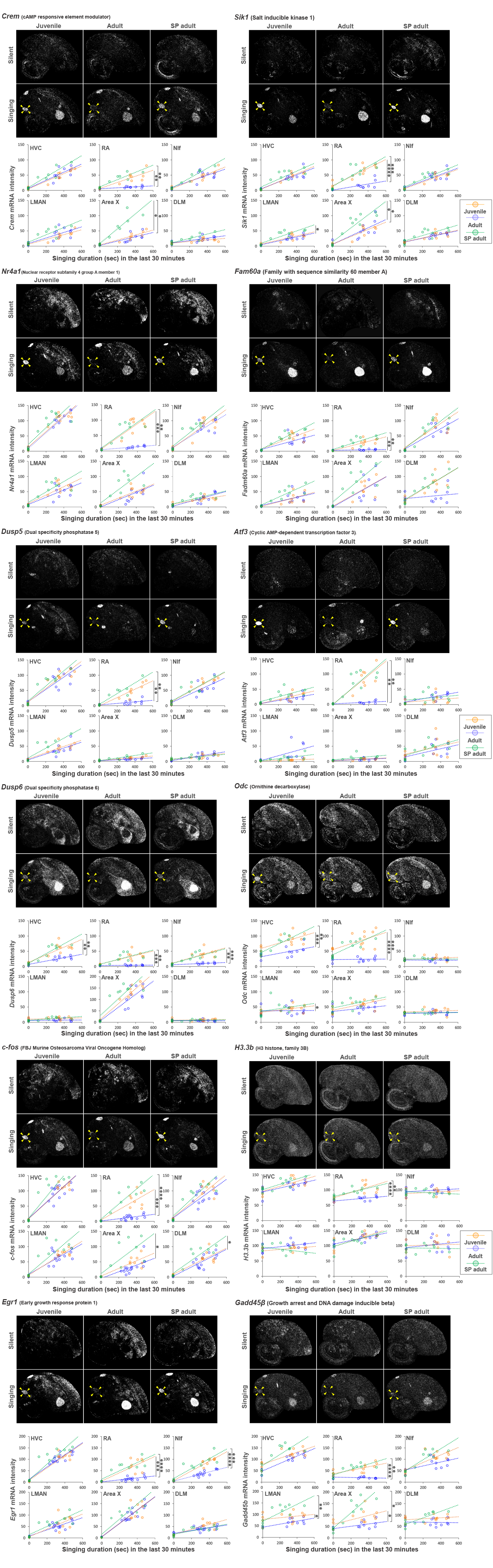

Supplement: S5 Fig — Expression patterns of Crem, Nr4a1, Sik1, Fam60a, Dusp5, Dusp6, Atf3, Odc, c-fos, Egr1, H3.3b, and Gadd45β in silent and singing conditions of juveniles, adults, and SP adults 1–2 days after release from singing prevention. Expression dynamics of the genes after singing in the song nuclei (HVC, RA, NIF, LMAN, Area X, and DLM) in juveniles (orange), adults (purple), and SP adults (green). Lines represent the linear approximation curve. (*p < 0.01, **p < 0.001, ***p < 0.0001, ****p < 0.00001; ANCOVA with Bonferroni correction). Supporting data can be found in S4 Data. ANCOVA, analysis of covariance; DLM, dorsal lateral nucleus of the medial thalamus; LMAN, lateral magnocellular nucleus of the anterior nidopallium; NIF, interfacial nucleus of the nidopallium; RA, robust nucleus of the arcopallium; SP, singing-prevented. (TIF) [file pbio.2006537.s005.tif]

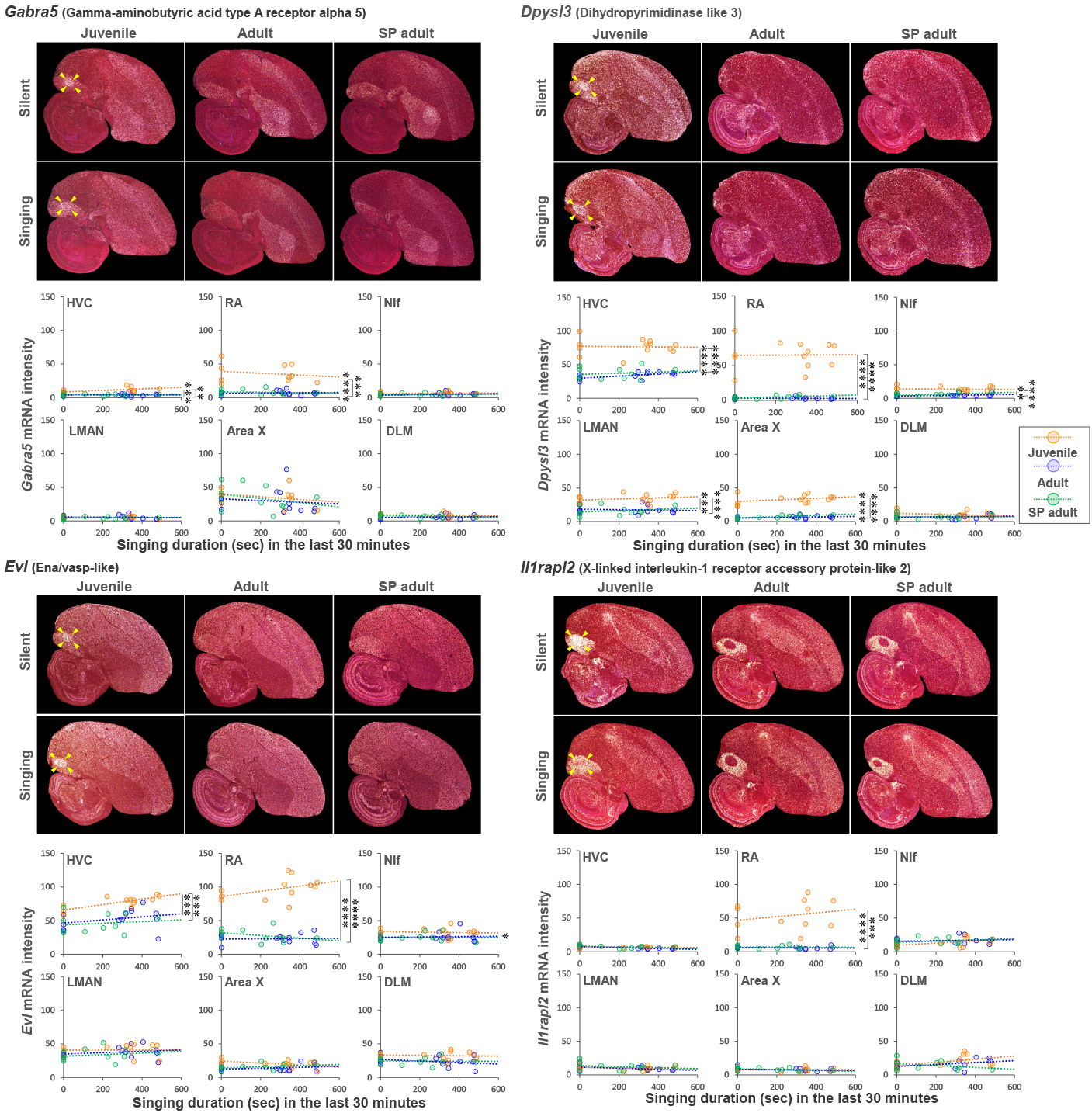

Supplement: S6 Fig — Expression patterns of Gabra5, Evl, Dpysl3, and Il1rapl2 from RA Cluster II genes under silent and singing conditions in juveniles, adults, and SP adults 1–2 days after release from singing prevention. Expression dynamics of the genes in the song nuclei (HVC, RA, NIF, LMAN, Area X, and DLM) in juveniles (orange), adults (purple), and SP adults (green). Lines represent the linear approximation curve. (*p < 0.01, **p < 0.001, ***p < 0.0001, and ****p < 0.00001; ANCOVA with Bonferroni correction). Supporting data can be found in S4 Data. ANCOVA, analysis of covariance; DLM, dorsal lateral nucleus of the medial thalamus; LMAN, lateral magnocellular nucleus of the anterior nidopallium; NIF, interfacial nucleus of the nidopallium; RA, robust nucleus of the arcopallium; SP, singing-prevented. (TIF) [file pbio.2006537.s006.tif]

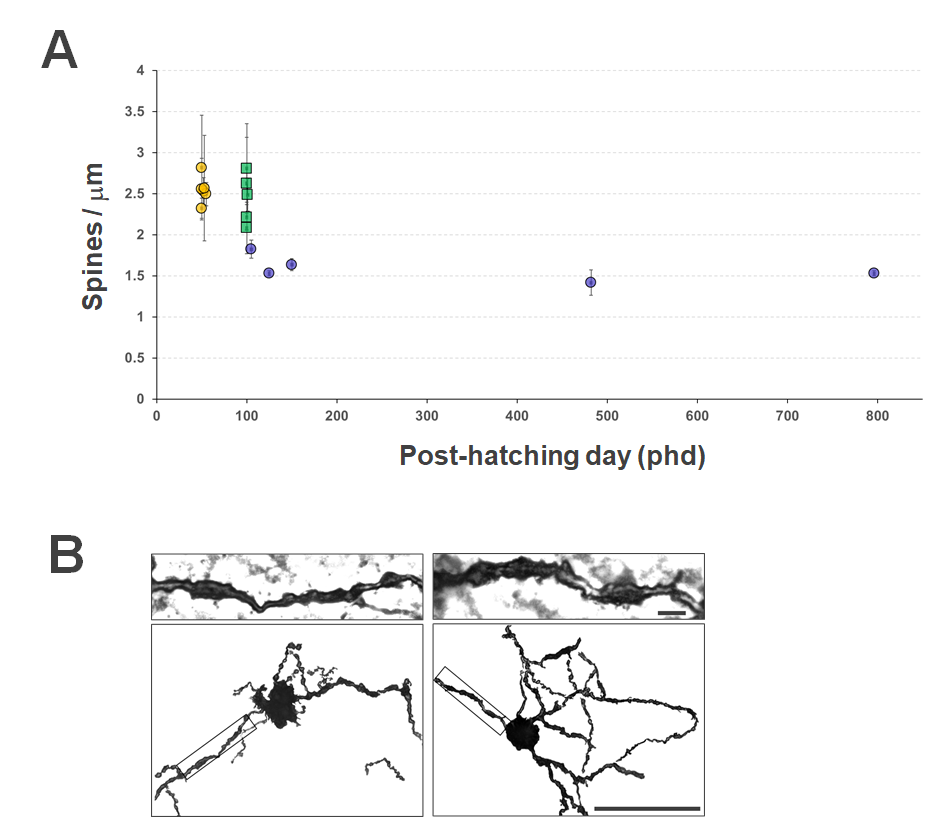

Supplement: S7 Fig — (A) Developmental changes in the dendritic spine density of RA projection neurons in juveniles (n = 6: orange), adults (n = 5: purple), and SP adults at 1–2 days after release from singing prevention (n = 5: green). Error bars: SD. Supporting data can be found in S6 Data. (B) Golgi-stained RA interneurons in normal adult (left) and SP (right) birds. Scale bars = 5 μm (upper) and 50 μm (lower). RA, robust nucleus of the arcopallium; SP, singing-prevented. (TIF) [file pbio.2006537.s007.tif]

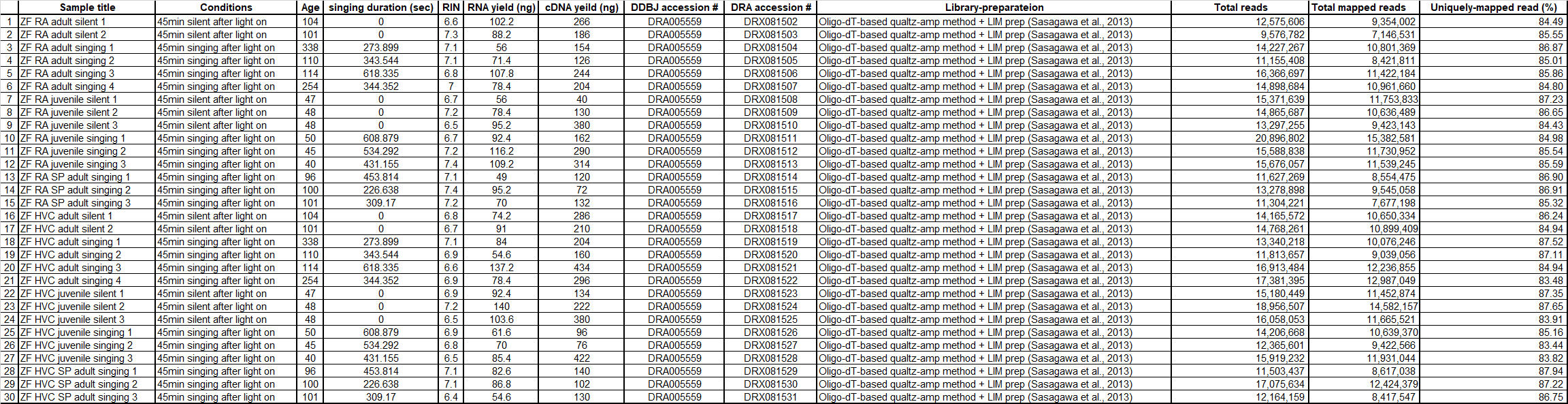

Supplement: S1 Table — RNA-seq, RNA sequencing. (TIF) [file pbio.2006537.s008.tif]

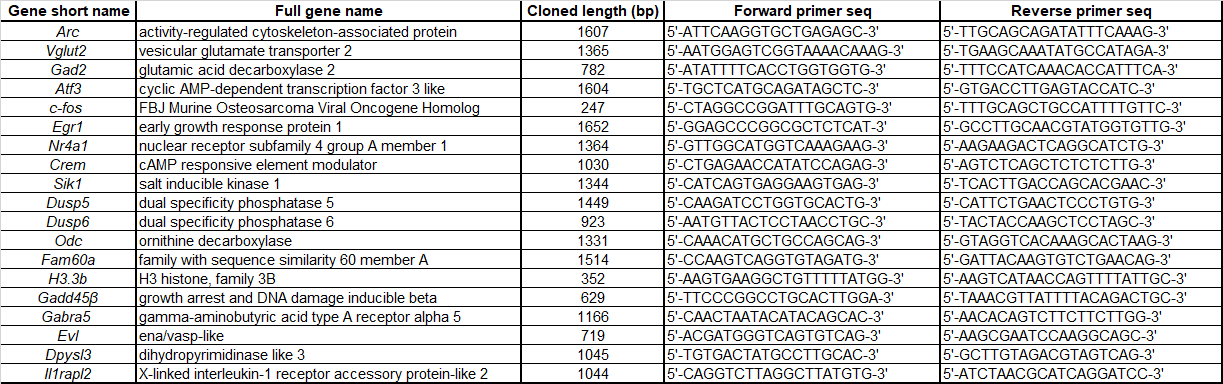

Supplement: S2 Table — (TIF) [file pbio.2006537.s009.tif]
